# Supplementary material for: Flow parallel synthesizer for multiplex synthesis of aryl diazonium libraries via efficient parameter screening
Source: Commun Chem. 2021 Apr 15;4:53. doi: 10.1038/s42004-021-00490-6 (PMC9814388; doi:10.1038/s42004-021-00490-6)

Flow Parallel Synthesizer for Multiplex Synthesis of Aryl Diazonium Libraries via Efficient Parameter Screening

Gwang-Noh Ahn‡, Brijesh M. Sharma‡, Santosh Lahore, Se-Jun Yim, Shinde Vidyacharan and Dong-Pyo Kim*

Author information

Affilations

Center for Intelligent Microprocess of Pharmaceutical Synthesis, Department of Chemical Engineering, Pohang University of Science and Technology (POSTECH), Pohang 790-784, Republic of Korea

Gwang-Noh Ahn‡, Brijesh M. Sharma‡, Santosh Lahore, Se-Jun Yim, Shinde Vidyacharan and Dong-Pyo Kim*

‡ These authors contributed equally to this work.

CORRESPONDING AUTHOR

* Dong-Pyo Kim, E-mail: dpkim@postech.ac.kr

Electronic Supplementary Information

Table of contents:

1) Manufacturing and assembly of flow parallel synthesizer ……………………………….. S3

2) Computational fluid dynamics on pressure gradient in flow parallel synthesizer …..…..….. S4

3) Temperature profile by IR photo imaging ……………..……………………………………… S5

4) Supplementary Notes 1 : Computational and experimental fluid dynamics on flow distribution performance ……………………………………………………………………………… S6-S11

5) Supplementary Notes 2 : Synthesis in various reactors …...……….……………………S12-S16

6) Multiplex and simultaneous optimization of compound libraries …………………….. S17-S18

7) Comparison of the latest chemical reaction screening platforms ……………………..…. S19

8) Supplementary Notes 3 : Spectral data of all synthesized compounds………………….S20-S23

9) Supplementary References……………………………………………………………….. S24

10) NMR copies of all synthesized compounds (3a-3x) ………………………………….. S25-S68

1) Manufacturing and assembly of flow parallel synthesizer

Supplementary Figure 1: Schematic of cross-sectional layout drawing with detailed dimensions of metal-based parallel flow platform.

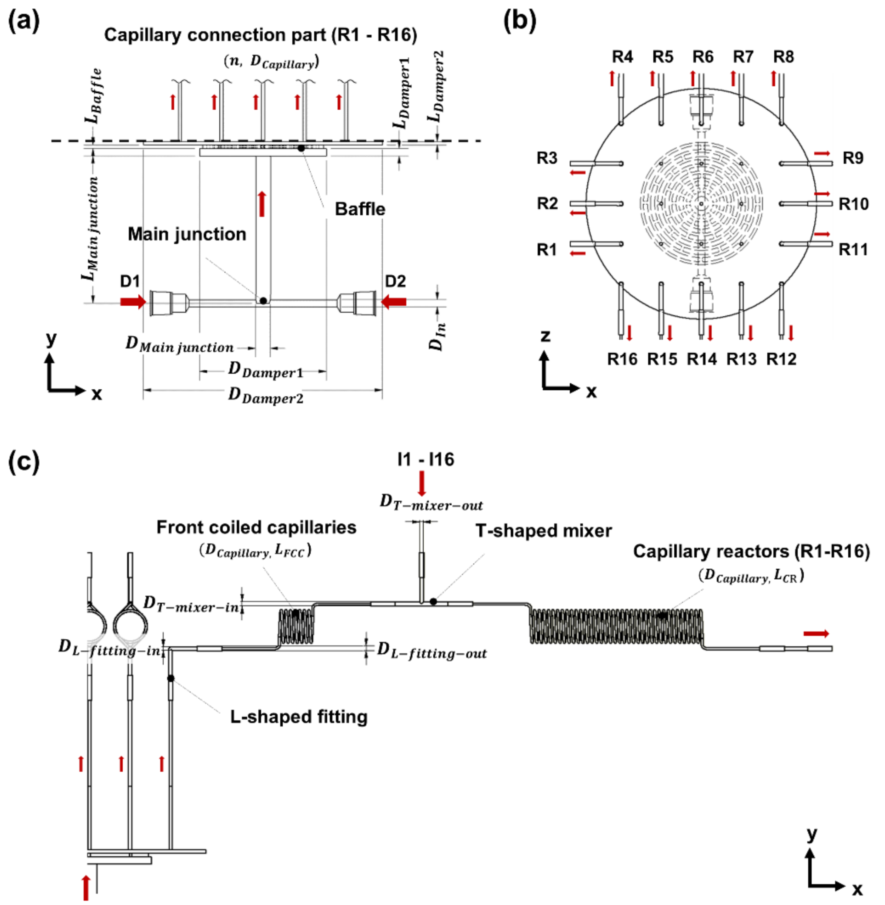

Design parameter	Design condition	Design parameter	Design condition
D_{In}	2.4 mm	n	16
$D_{Main\ junction}$	5 mm	$D_{L-fitting-in}$	1.27 mm
$D_{Damper1}$	81 mm	$D_{L-fitting-out}$	1.59 mm
$D_{Damper2}$	43 mm	$D_{T-mixer-in}$	1.27 mm
$L_{Main\ junction}$	50.11 mm	$D_{T-mixer-out}$	1.59 mm
L_{Baffle}	1.25 mm	$D_{Capillary}$	0.75 mm
$L_{Damper1}$	2.5 mm	L_{FCC}	350 mm
$L_{Damper2}$	1.15 mm	L_{CR}	1,500 mm

(a) the x–y plane of flow distributor structure, **(b)** the x–z plane with 16 capillary exits (R1~R16) at the top part, baffle structure at the middle part, and **(c)** the x-y plane of a typical capillary reactor where main species is merged with building block at T-mixer.

2) Computational fluid dynamics on pressure gradient in flow parallel synthesizer

Supplementary Figure 2: Calculated pressure gradient across x-y plane on the inlet of the flow parallel synthesizer, depending on different dimensions of front coiled capillaries

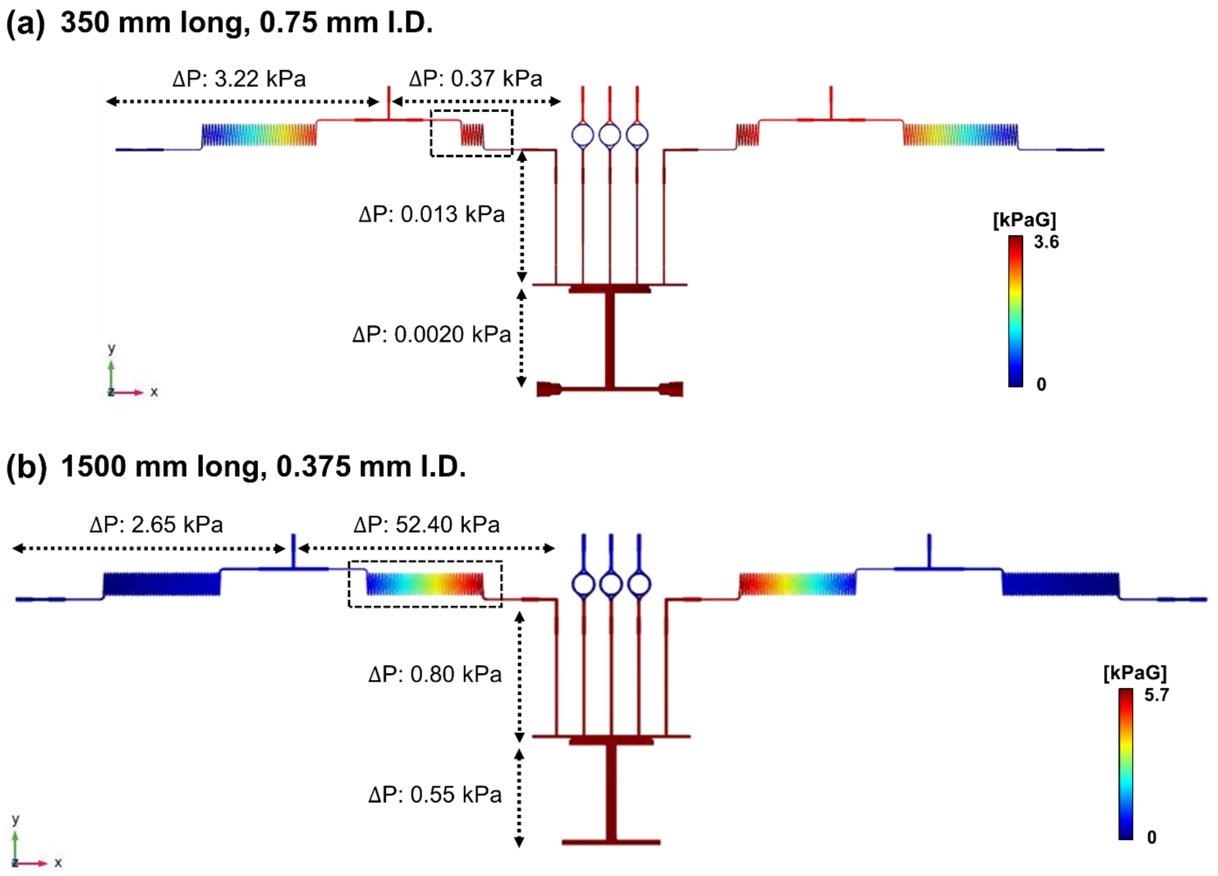

Assumed the DMSO is fed through I1-I16 at 0.33 mL/min, through D1 and D2 at a flow rate of 5.28 mL/min. Other geometric conditions are shown in **Supplementary Fig. 1**.

3) Temperature profile by IR photo imaging

Supplementary Figure 3: IR photo images of coiled reactors in a parallel flow synthesizer.

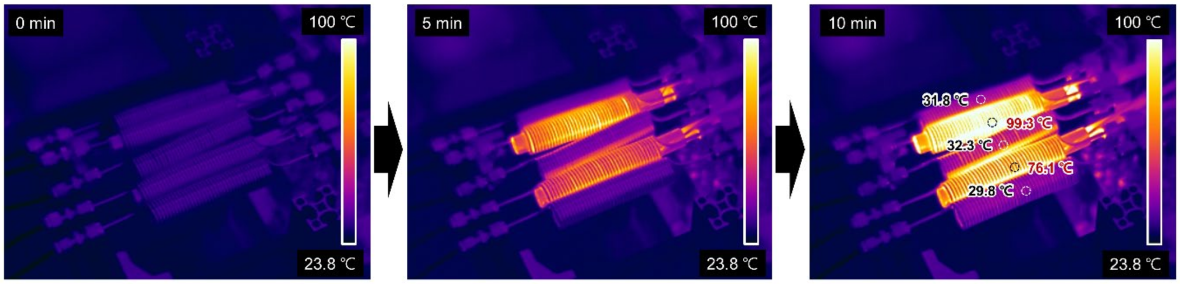

IR photo images (top view) to confirm the controlled heating of only two coiled reactors in the parallel flow synthesizer. Temperature was set at 100 °C (**R13**) and 75°C (**R15**) by using a Proportional-Integral-Differential (PID) based temperature controller, thermocouple and heating rod. **R13** and **R15** were reached to the set points steadily in a short time (10 min), while other coiled microreactors nearby stably maintained at room temperature. Note that the use of adequate insulation filled into the space (1 mm) between reactors enables reliable temperature control.

4) Supplementary Notes 1 : Computational and experimental fluid dynamics on flow distribution performance

First, the CFD numerical analysis and experiment for flow distribution were performed assuming a situation where uniform distribution of flow to all capillaries is required for screening the yield of individual reactions under the various residence time conditions. The DMSO solution was injected at the flow rate of six cases (10.56, 5.28, 2.64, 1.06, 0.53 and 0.35 mL/min) through the main inlets (**D1** or **D2** in **Figure 2**). In this case, the peristaltic pumps (**P1** to **P3**) were selectively detached. The boundary conditions for **I1** to **I16** were set so that flows including building blocks were joined at flow rates of six cases (0.66, 0.33, 0.17, 0.066, 0.033 and 0.022 mL/min) through T-mixers. Since the volume of each capillary was 0.662 mL, the residence time of each capillary became 30, 60, 120, 300, 600 and 900 sec. Finally, flow uniformity was quantified by checking the outlet flow rate from 16 individual capillaries and obtaining the MF values. In both numerical and experimental results, the MF value was sufficiently low less than 1, 4% respectively[1] (**Supplementary Fig. 4** and **Supplementary Table 1)**. In addition, the unique design of our system allowed even distribution of diazonium salt solution in DMSO, as shown in **Supplementary Fig. 5.**

The clogging as a chronic problem of microreactor may occur at some non-optimized organic reactions. The clogging occurred in either of capillaries (out of **R12** ~ **R16**) was investigated how it affected other capillaries. As a result, MF in the rest of capillaries, except the clogged capillaries, as shown in **Supplementary Fig. 6** and **Supplementary Table 2**, was less than 3 %, either numerically or experimentally, showing uniform flow distribution behavior.

Next, for multiplex and simultaneous synthesis of the compound libraries at optimized conditions, the simulation was carried out by giving the change in residence time into several capillaries. It was assumed that the solution was injected at a flow rate of 5.14 mL/min through the main inlet (**D1** or **D2** in **Figure 2**). To reflect the change of the flow rate in the three capillaries through peristaltic pumps (**P1** to **P3**), flow rate 0.033, 0.17 and 0.66 mL/min were set as the boundary condition of outlet flow before the T-mixers for these three outlet capillaries. The building block flow for the three capillaries (**I1** to **I3**) also set the flow rate to 0.033, 0.17 and 0.66 mL/min. In the case of other capillaries, boundary conditions were set for **I4** to **I16** so that a flow rate of 0.33 mL/min would merge through each T-mixer. Finally, the flow distribution was quantified by checking the outlet flow rate from the remaining 13 capillaries, excluding 1 to 3 capillaries.

(**Supplementary Fig. 7**) Through numerical analysis, the MF calculated from the flow distribution at the outlets other than outlets 1 to 3 was less than 1 %. Through this, it was seen that even when the flow rates of capillaries 1 to 3 were selectively changed through peristaltic pumps (**P1** to **P3**), the flow distribution in other capillaries was maintained uniformly. In addition, the experimentally confirmed MF was less than 4 %, indicating that the uniformity of the flow distribution was sufficient.

In addition, numerical analysis on the dimensional effect of front coiled capillaries (350 mm long / 0.75 mm I.D., 1500 mm long / 0.375 mm I.D.) was thoroughly investigated to compare decoupling of the main flow at all different flow rates of building blocks in the system (see **Supplementary Fig. 8**). The longer and narrower coiled capillary, the more clearly decouples the flow of the main species stream from the flow of the building block species.

Supplementary Figure 4: Comparison of experimental (black line) and numerical (red line) flow distribution performance of 16 outlets under various flow rate conditions.

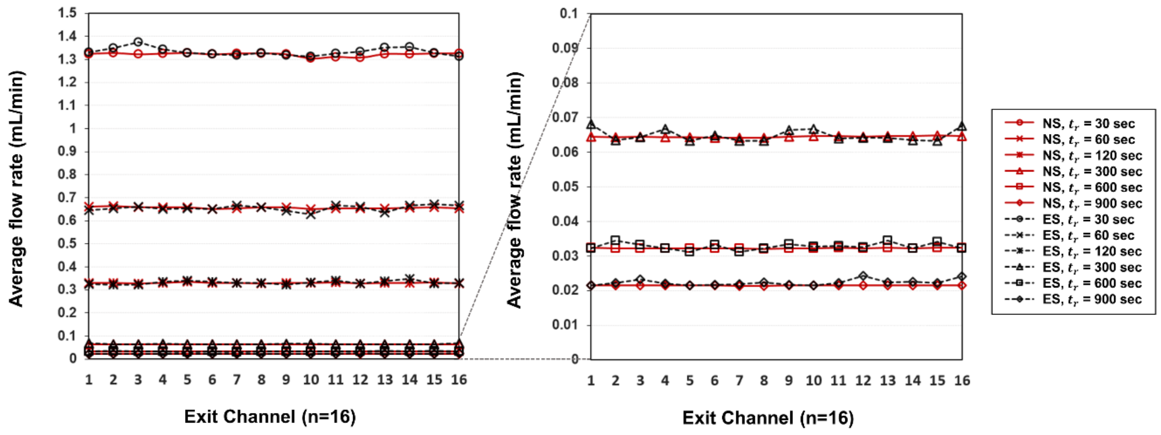

DMSO was injected through main inlet (**D1** or **D2**) at flow rates of 10.56, 5.28, 2.64, 1.06, 0.53 and 0.35 mL/min, respectively. The flow of building blocks entered through inlets **I1** to **I16** at flow rates of 0.66, 0.33, 0.17, 0.066, 0.033 and 0.022 mL/min in each case. The MF values obtained through experimental and numerical studies were summarized in **Table S1.**

Supplementary Table 1: MF values obtained from Supplementary Fig. 2.

Flow rate (mL/min)	D1 or D2	10.56	5.28	2.64	1.06	0.53	0.35
	I1 ~ I16	0.66	0.33	0.17	0.066	0.033	0.022
Residence time (s)		30	60	120	300	600	900
Numerical study, MF (%)		0.54	0.59	0.53	0.51	0.22	0.25
Experimental Study, MF (%)		1.27	1.85	2.27	2.61	3.06	3.86

Supplementary Figure 5: 16 sample vials collected from R1~R16 for experimental confirmation of uniform flow distribution.

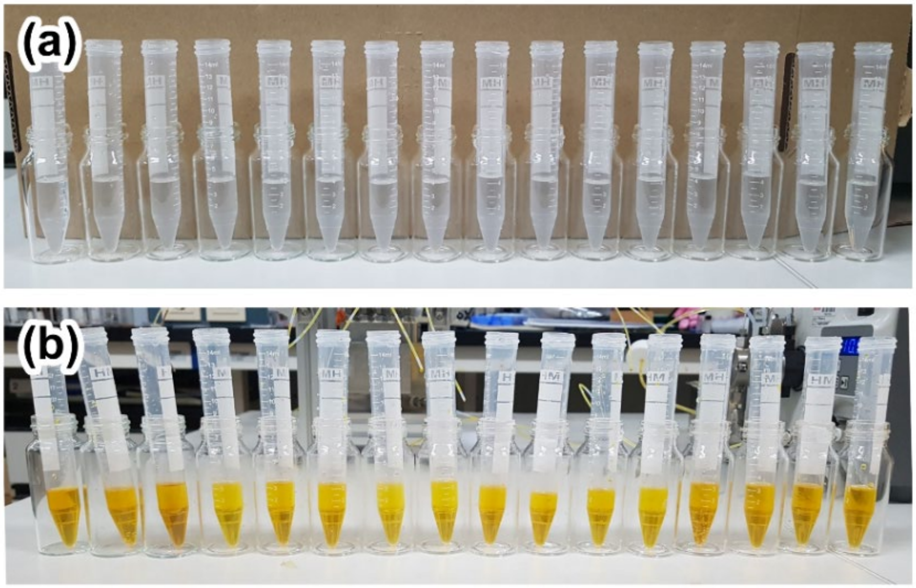

The solution or the reagent was injected at flow rate 10.56 mL/min through **D1** and **D2**. (a) only DMSO solvent, (b) 0.77 M diazonium salt solution in DMSO.

Supplementary Figure 6: Experimental and numerical analysis of uniform flow distribution on various clogging cases, by adjusting the main flow rate with no pausing the entire system.

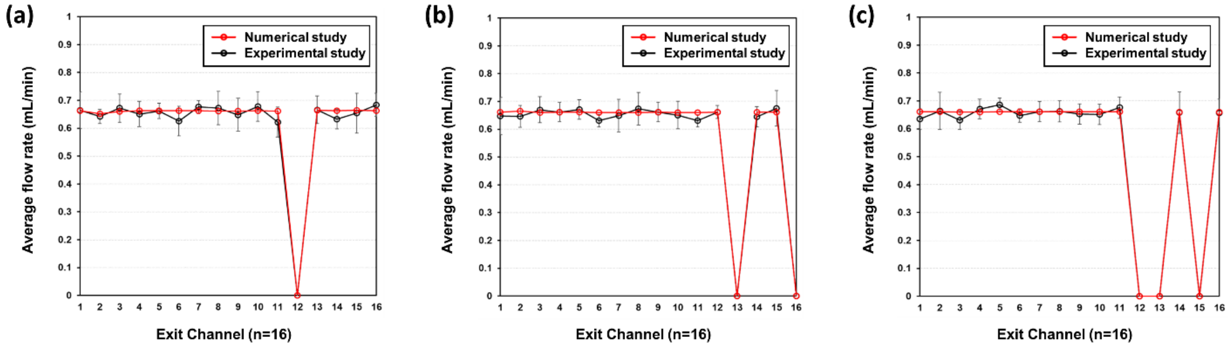

Cases	Number of reactors (clogged/unclogged)	Clogged reactors	Main flow rate to be adjusted	Distributed main flow rates	Building block flow rates
normal	0/16	-	5.28 ml/min	0.33 ml/min	0.33 ml/min
(a)	1/15	R12	4.95 mL/min (5.28 ml/min – 0. 33 ml/min)*	0.33 ml/min	0.33 ml/min
(b)	2/14	R13, R16	4.62 mL/min (5.28 ml/min – 0. 66 ml/min)	0.33 ml/min	0.33 ml/min
(c)	3/13	R12, R13, R15	4.29 mL/min (5.28 ml/min – 0. 99 ml/min)	0.33 ml/min	0.33 ml/min

* 0.33 ml/min = 6.3 % of 5.28 ml/min

The clogging was intentionally caused by capping the outlet of the T-mixer to block the flow. Experimental results represent the mean values and deviations of 3 runs. **(a)** On clogging of single capillary (**R12),** the main flow rate **(D1+D2)** was reduced to 4.95 mL/min from initial 5.28 mL/min to maintain the flow rate 0.33 mL/min at the non-clogged reactors. **(b)** On clogging of two capillaries **(R13, R16),** the **(D1+D2)** was reduced to 4.62 mL/min to maintain 0.33 mL/min at the non-clogged reactors. **(c)** On clogging of three capillaries (**R12**, **R13, R15),** the **(D1+D2)** was reduced to 4.29 mL/min to maintain 0.33 mL/min at the non-clogged reactors. The experimental and numerical MF values were summarized in **Supplementary Table 2.**

Supplementary Table 2: MF values obtained from Supplementary Fig. 4, calculated excluding the clogged channels.

Case	(a)	(b)	(c)
**Numerical study, MF (%)**	0.47	0.22	0.04
**Experimental Study, MF (%)**	2.96	2.25	2.31

Supplementary Figure 7: Uniform flow of main species in 13 reactors (R4-R16) when the other 3 reactors (R1-R3) were injected at different flow rates (0.033, 0.17, 0.66 mL/min) by peristaltic pumps.

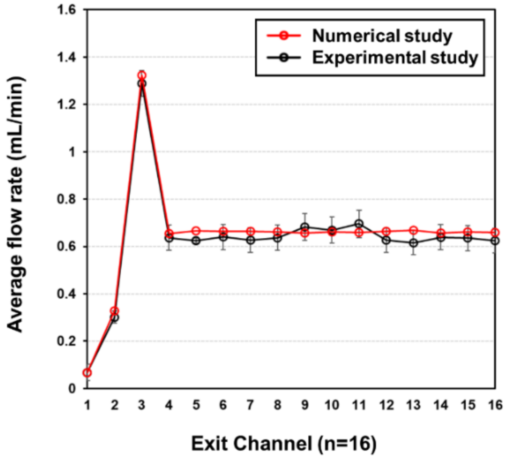

DMSO was injected through **D1** and **D2** at a flow rate of 5.14 mL/min, and also introduced through second inlets at **I1-I3** (0.033, 0.17, 0.66 mL/min) to become 1:1 merging ratio. For the remaining reactors **R4-R16**, the numerical MF value was 0.57 %, that was lower than experimental MF value of 3.37 %.

Supplementary Figure 8: **Simulated main stream flows at all different flow of building blocks, depending on front coiled capillaries with different dimensions.**

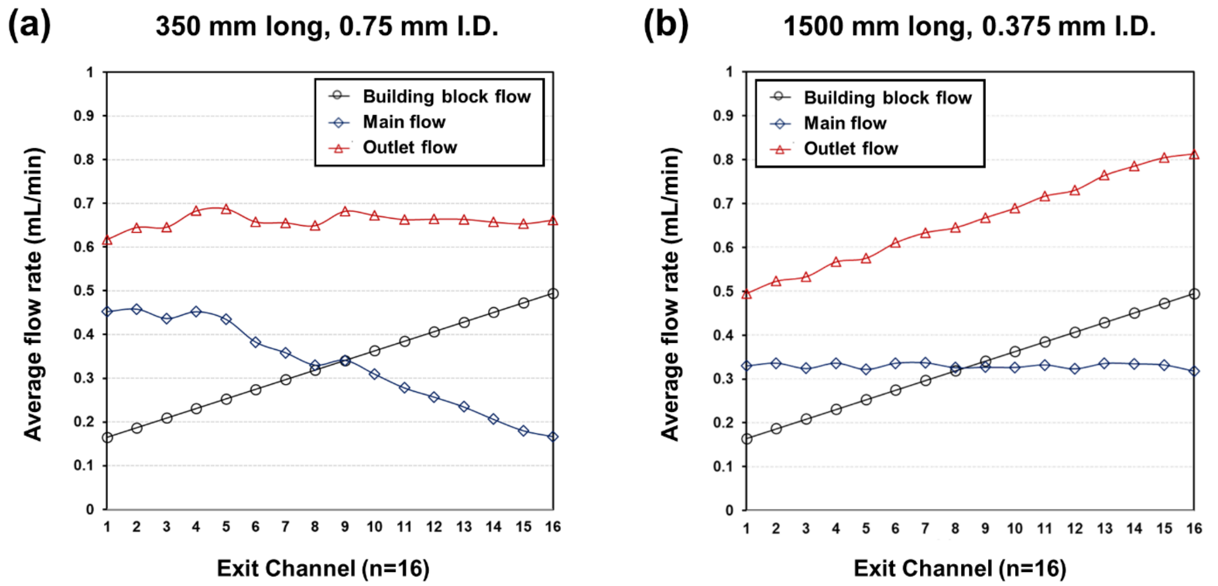

Assumed the DMSO is fed through **I1-I16** at all different flow rates that are linearly varied from 0.33 mL/min (as employed at actual chemical synthesis in **Figure 6**) with ±50% difference (0.165 ~ 0.495 ml/min), while the DMSO is injected through **D1** and **D2** at a flow rate of 5.28 mL/min. As a result, in case **(a)**, at low pressure in the system, the main flow rate is varied in inverse proportional to the change along the building block flow rates in I1-I16, then the outlet flow rates becomes almost constant. In the case of **(b)**, at considerably high pressure in the system, the distribution of the main flow is constant regardless of the difference among the building block flows, i.e. the main flow and outlet flow are decoupled. For both cases, the MF value of the main flows and the MF value at the outlets are **(a)** 30.7% and 2.6% **(b)** 1.8% and 15.7%, respectively. Other geometric conditions are shown in **Supplementary Fig. 1**

5) Supplementary Notes 2 : Synthesis in various reactors

A. Synthesis of starting reagents in batch

- **General procedure for the synthesis of aryl diazonium tetrafluoroborates salt -**

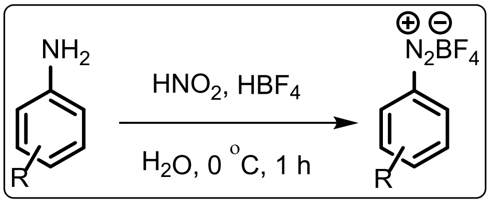

The appropriate aniline (214.75 mmole, 20 g, 1 equiv.) was dissolved in a mixture of 86 mL of distilled water and 57 mL of 48 wt% hydrofluoroboric acid. After cooling the reaction mixture to 0 °C using ice bath, the sodium nitrite (15.6 g in 32 mL) solution was added dropwise in 5 min interval of time. The resulting mixture was stirred for 1 h and the precipitate was collected by filtration and re-dissolved in minimum amount of acetone. Diethyl ether was added until precipitation of diazonium tetrafluoroborate, which is filtered, washed several times with diethyl ether and dried under vacuum[2].

- **General procedure for the synthesis of imidazopyridine and imidazothiazole -**

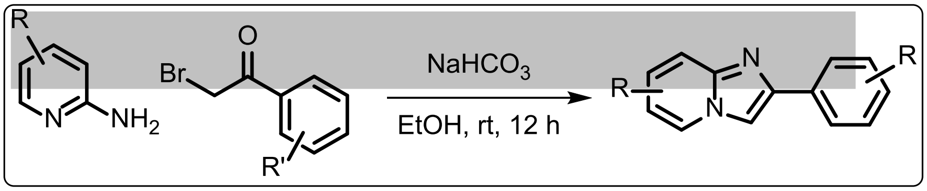

A solution of 2-aminopyridine/ 2-aminothiazole (5 mmol, 1 equiv) and bromomethyl ketone 30 (5 mmol, 1 equiv) in EtOH (30 mL) was heated under reflux for 16 h and the progress of the reaction was monitored using TLC. The solvent was removed under reduced pressure, and saturated solution of $NaHCO_3$ (30 mL) was added to the remaining solid. The mixture was then extracted with EtOAc (30 mL, ×3), and the organic layers were combined, dried over Na_2SO_4. The concentrated crude product was dried overnight under vacuum followed by trituration using (4:1) EtOAc : *n*-Hexane to get the prude imidazopyridine/ imidazothiazole which was directly used in the next step[3].

B. Synthesis using diazonium salts in batch

- **S_NAr type reactions of diazonium in batch:**

➢ **Iodination reaction**

A diazonium salts (738 mg, 3.85 mmoles) was dissolved in 9 mL of DMSO. After cooling the reaction mixture to 20 °C, a solution of potassium iodide (640 mg, 3.85 mmoles) in 1 mL of H_2O was added dropwise with lead to the generation of nitrogen gas and the mixture was stirrer for 30 min. The progress of the reaction was monitored by GC. The product was then diluted with water and extracted three times with ether. The organic layer was washed with brine, dried over Na_2SO_4 and evaporated under reduced pressure. The resulting compound was absorbed on silica gel and purified through column chromatography (*n*-pentane/*n*-hexane, 1:1) to furnish iodobenzene as a colorless liquid in 70 % yield.

➢ **Chlorination reaction**

A diazonium salts (738 mg, 3.85 mmoles) was dissolved in 8 mL of DMSO. After cooling the reaction mixture to 20 °C, a solution of and CuCl (518 mg, 3.85 mmoles) in 2 mL of HCl was added dropwise, leading to generation of nitrogen gas and the reaction mixture was stirrer for 30 min. The progress of the reaction was monitored using GC. The product was then diluted with water and extracted three times with ether. The organic layer was washed with brine, dried over Na_2SO_4 and evaporated under reduced pressure. The resulting compound was absorbed on silica gel and purified through column chromatography (*n*-pentane/*n*-hexane, 1:1) to furnish chlorobenzene as a colorless liquid in 64 % yield.

➢ **Azidation reaction**

A diazonium salts (738 mg, 3.85 mmoles) was dissolved in 9 mL of DMSO. After cooling the reaction mixture to 20 °C, a solution of sodium azide (250 mg, 3.85 mmoles) in 1 mL of H_2O was added dropwise with lead to the generation of nitrogen gas and the mixture was stirrer for 30 min. The progress of the reaction was monitored by GC. The product was then diluted with water and extracted three times with ether. The organic layer was washed with brine, dried over Na_2SO_4 and evaporated under reduced pressure. The resulting compound was absorbed on silica gel and

purified through column chromatography (*n*-pentane/*n*-hexane, 1:1) to furnish azidobenzene as a pale-yellow liquid in 89 % yield.

- **Synthesis of diaryl sulfides**

A diazonium salts (738 mg, 3.85 mmoles) was dissolved in 4 mL of DMSO. After cooling the reaction mixture to 20 ºC, a solution of *p*-thiocresol (478 mg, 3.85 mmoles) and NaOH (154 mg, 3.85) dissolved in 6 mL of DMSO:H_2O (2:1) was added dropwise with lead to the generation of nitrogen gas and the mixture was stirrer for 2 h. The progress of the reaction was monitored by TLC. The product was then diluted with water and extracted three times with ether. The organic layer was washed with brine, dried over Na_2SO_4 and evaporated under reduced pressure. The resulting compound was absorbed on silica gel and purified through column chromatography (*n*-hexane/ethylacetate, 98:2) to furnish diaryl sulfides as a colorless liquid in 60 % yield.

- ***α*-Arylation of furan**

A diazonium salts (738 mg, 3.85 mmoles) was dissolved in 9 mL of DMSO. After cooling the reaction mixture to 20 ºC, a solution of furan (5.6 mL, 77 mmoles) and 4-aminomorpholine (0.193 mmoles, 5 mol%) dissolved in 10 mL of DMSO was added dropwise with lead to the generation of nitrogen gas and the mixture was stirrer for 30 min. The progress of the reaction was monitored by TLC. The product was then diluted with water and extracted three times with ether. The organic layer was washed with brine, dried over Na_2SO_4 and evaporated under reduced pressure. The resulting compound was absorbed on silica gel and purified through column chromatography (*n*-hexane/ethylacetate, 98:2) to furnish *α*-arylated furan as a colorless liquid in 55 % yield.

- **Photochemical reactions for *α*-Arylation of furan**

A diazonium salts (738 mg, 3.85 mmoles) and furan (38.5 mmoles) were dissolved in 10 mL DMSO to which was added eosin Y (0.193 mmoles, 5 mol%) and the reaction mixture was stirrer for 2 h under green LED and the progress of the reaction was monitored using TLC. The product was then diluted with water and extracted three times with ether. The organic layer was washed with brine, dried over Na_2SO_4 and evaporated under reduced pressure. The resulting compound was absorbed on silica gel and purified through column chromatography (*n*- hexane/ethylacetate, 98:2) to furnish *α*-arylated furan as a colorless liquid in 61 % yield.

➢ Arylation of imidazopyridine / imidazothiazole in batch

A diazonium salts (738 mg, 3.85 mmoles) and imidazopyridine / imidazothiazole (2.55 mmoles) was taken together and dissolved in 10 mL of DMSO and the reaction mixture was stirrer at room temperature for 8 h and the progress of the reaction was monitored using TLC. The product was then diluted with water and extracted three times with ethyl acetate. The organic layer was washed with brine, dried over Na_2SO_4 and evaporated under reduced pressure. The resulting compound was absorbed into silica gel and purified through column chromatography (*n*-hexane/ethylacetate, various ratios).

➢ Azo-coupling of diazonium salts and *β*-naphthol in batch

A diazonium salts (6.375, 5.1, 3.825, 3.1875 and 2.55 mmoles each) was dissolved in 10 mL DMSO in 5 different vials. After cooling the reaction mixture to 20 °C, solution of *β*-naphthol and NaOH (6.375, 5.1, 3.825, 3.1875 and 2.55 mmoles each) dissolved in 10 mL of DMSO:H_2O (9:1) were added dropwise and the mixture was stirrer for 1 h and the progress of the reaction was monitored using TLC. The product was then diluted with water and extracted three times with ethyl acetate. The organic layer was washed with brine, dried over Na_2SO_4 and evaporated under reduced pressure. The resulting compound was absorbed into silica gel and purified through column chromatography (*n*-hexane/ethylacetate 98:2) to give reddish orange solid in 77, 79, 78, 76 and 75% yields respectively.

C. General procedure for S_NAr type reactions of diazonium in a single capillary:

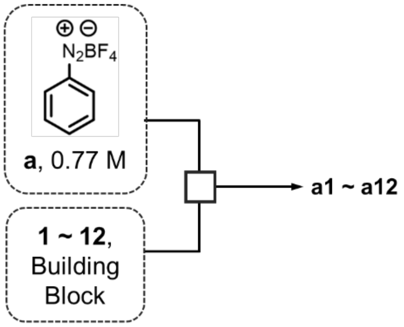

Building blocks

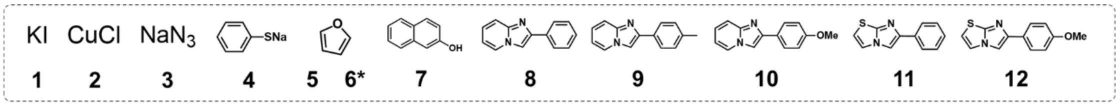

6* Photo-reaction

A solution of the diazonium (0.77 M) and other building blocks were prepared as per general procedure **C.** Both stock solutions of diazonium and building blocks were transferred respectively to two 10 mL NORM-JECT plastic syringes. These stock solutions of diazonium and building blocks were pumped into a PTFE capillary tube (L = 1500 mm, 1/16' O.D., 0.75 mm I.D.) using two syringe pumps at flow rates (**6**: 0.033 mL/min, **2**: 0.17 mL/min, **1**: 0.66 mL/min and all other building blocks (**3-5** and **7-12**) have 0.33 mL/min; flow rates ratio of diazonium and building block solutions = 1:1) through T-mixer to render the required residence times as shown in **Supplementary Table 3** (under heading of capillary yield) at room temperature. At a steady state the samples were collected for each individual reactions and quenched using water and the workup, purification procedure was similar to the one described in batch (general procedure **B)**.

6) Multiplex and simultaneous optimization of compound libraries

Supplementary Table 3: Summary for optimization of 96 reaction conditions in a multiplex manner.

Entry	Rx. Type	Reactor #	Conc. of building block	Reaction time	Product	Yield, % [e]	Batch Yield, % [e]	Capillary Yield, % [e]
**1**	a1	**R3**	**0.77 M**	**t_r = 30 s**	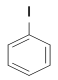	**65**	72<br>30 min	75<br>30 s
2				t_r = 60 s		61		
3				t_r = 120 s		63		
4				t_r = 300 s		62		
5				t_r = 600 s		60		
6				t_r = 900 s		58		
7	a2	**R2**	**0.77 M**	t_r = 30 s	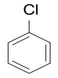	30	64<br>30min	66<br>120 s
8				t_r = 60 s		45		
**9**				**t_r = 120 s**		**57**		
10				t_r = 300 s		56		
11				t_r = 600 s		53		
12				t_r = 900 s		46		
13	a3	**R9**	**0.77 M**	t_r = 30 s	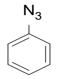	70	89<br>30 min	91<br>60 s
**14**				**t_r = 60 s**		**86**		
15				t_r = 120 s		87		
16				t_r = 300 s		85		
17				t_r = 600 s		82		
18				t_r = 900 s		79		
19	a4	**R10**	**0.77 M**	t_r = 30 s	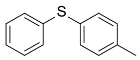	48	60<br>2 h	64<br>60 s
**20**				**t_r = 60 s**		**55**		
21				t_r = 120 s		54		
22				t_r = 300 s		53		
23				t_r = 600 s		51		
24				t_r = 900 s		47		
25	a5 [c]	**R11**	**neat**	t_r = 30 s	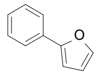	54	55<br>30 min	62<br>60 s
**26**				**t_r = 60 s**		**60**		
27				t_r = 120 s		61		
28				t_r = 300 s		60		
29				t_r = 600 s		58		
30				t_r = 900 s		56		
31	a6 [d]	**R1**	**7.7 M**	t_r = 30 s	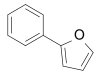	n.r	61<br>2 h	55<br>600 s
32				t_r = 60 s		6		
33				t_r = 120 s		15		
34				t_r = 300 s		32		
**35**				**t_r = 600 s**		**54**		
36				t_r = 900 s		45		
37	a7	**R12**	**0.64 M**	t_r = 30 s	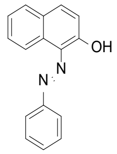	**Clog**	77<br>1 h	**Clog**
38				t_r = 60 s				
39				t_r = 120 s				
40				t_r = 300 s				
41				t_r = 600 s				
42				t_r = 900 s				
43	a7	**R13**	**0.51 M**	t_r = 30 s	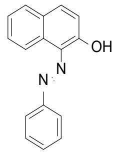	**Clog**	79<br>1 h	**Clog**
44				t_r = 60 s				
45				t_r = 120 s				
46				t_r = 300 s				
47				t_r = 600 s				
48				t_r = 900 s				

Entry	Rx. Type	Reactor #	Conc. of building block	Reaction time	Product	Yield, % [e]	Batch Yield, % [e]	Capillary Yield, % [e]
49	a7	**R14**	**0.38 M**	t_r = 30 s	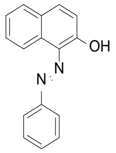	57	78<br>1 h	79<br>60 s
50				t_r = 60 s		**74**		
51				t_r = 120 s		76		
52				t_r = 300 s		71		
53				t_r = 600 s		**Clog**		
54				t_r = 900 s		**Clog**		
55	a7	**R15**	**0.32 M**	t_r = 30 s	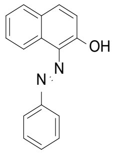	57	76<br>1 h	78<br>60 s
**56**				**t_r = 60 s**		**73**		
57				t_r = 120 s		71		
58				t_r = 300 s		70		
59				t_r = 600 s		**Clog**		
60				t_r = 900 s		**Clog**		
61	a7	**R16**	**0.26 M**	t_r = 30 s	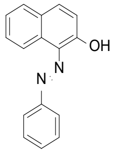	55	75<br>1 h	80<br>60 s
**62**				**t_r = 60 s**		**71**		
63				t_r = 120 s		72		
64				t_r = 300 s		71		
65				t_r = 600 s		**Clog**		
66				t_r = 900 s		**Clog**		
67	a8	**R4**	**0.51 M**	t_r = 30 s	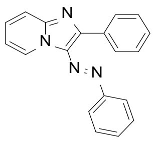	61	78<br>8 h	80<br>60 s
**68**				**t_r = 60 s**		**75**		
69				t_r = 120 s		76		
70				t_r = 300 s		75		
71				t_r = 600 s		70		
72				t_r = 900 s		69		
73	a9	**R5**	**0.51 M**	t_r = 30 s	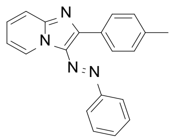	66	79<br>8 h	82<br>60 s
**74**				**t_r = 60 s**		**84**		
75				t_r = 120 s		83		
76				t_r = 300 s		80		
77				t_r = 600 s		78		
78				t_r = 900 s		76		
79	a10	**R6**	**0.51 M**	t_r = 30 s	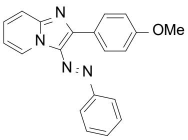	69	83<br>8 h	89<br>60 s
**80**				**t_r = 60 s**		**85**		
81				t_r = 120 s		87		
82				t_r = 300 s		85		
83				t_r = 600 s		81		
84				t_r = 900 s		80		
85	a11	**R7**	**0.51 M**	t_r = 30 s	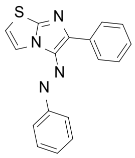	64	74<br>8 h	81<br>60 s
**86**				**t_r = 60 s**		**76**		
87				t_r = 120 s		75		
88				t_r = 300 s		77		
89				t_r = 600 s		71		
90				t_r = 900 s		72		
91	a12	**R8**	**0.51 M**	t_r = 30 s	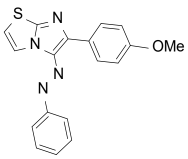	60	78<br>8 h	80<br>60 s
**92**				**t_r = 60 s**		**74**		
93				t_r = 120 s		76		
94				t_r = 300 s		75		
95				t_r = 600 s		71		
96				t_r = 900 s		68		

[a] Concentration of aryl-diazonium salt is 0.77 M and temperature of reaction is room temperature. [b] Residence time at each reactors. [c] 4-Aminomorpholine (5 mol%) was premixed with building block solution. [d] Eosin-Y (5 mol%) was premixed with building block solution. a 530 nm green LED is wrapped around the transparent PFA capillary. [e] Isolated Yield.

7) Comparison of the latest chemical reaction screening platforms

Supplementary Table 4: A comparison of the latest platform for High-Throughput Experimentation (HTE)

	Batch based	Flow based		
**Combinatorial chemistry**<br><br>Many substrate combinations<br><br>Small set of conditions	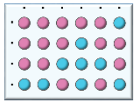<br>**Microwell-based screening**<br>• Platform to execute a large number of experiments with less effort per experiment.<br>• Quickly determine the optimal catalyst, reagent and solvent	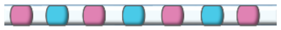<br>**Segment flow-based screening**<br>• Platform based on segment flow in microfluidic systems with high heat and mass transfer<br>• Optimize various reaction parameters as well as catalysts, reagents and solvents on a nano-micromole scale within sequentially flowing segments		
	Ref. : 4 - 8	Ref. : 9 - 12		
**Process chemistry**<br><br>Small substrate combinations<br><br>Many set of conditions	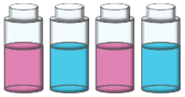<br>**Parallel synthesizer**<br>• Platform primarily used to explore the conditions of experiments requiring stability, such as oxygen or moisture intolerance polymerization.<br>• Simultaneously optimize the conditions of several reactions	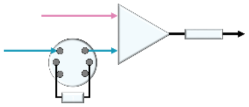<br>**Sequential flow synthesis**<br>• Flow-based screening device by sequential liquid injection device<br>• Perform and optimize various reactions sequentially	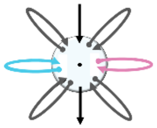<br>**Radial flow synthesis**<br>• Automated continuous flow platform arranged radially<br>• Perform and optimize multi-step reactions by combining continuous and sequential reactions	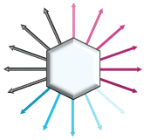<br>**Flow parallel synthesizer**<br>• Platform to perform multiple reactions simultaneously based on a sophisticated flow distributor<br>• Simultaneous search for optimal conditions for multiple single continuous reactions<br>• Sequential reaction time optimization and chemical library screening
	Ref. : 13 - 14	Ref. : 15	Ref. : 16	Our work

8) Supplementary Notes 3 : Spectral data of all synthesized compounds

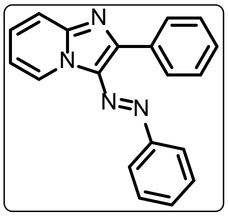

(*E*)-2-Phenyl-3-(phenyldiazenyl)imidazo[1,2-*a*]pyridine:

^{1}H NMR (500 MHz, $CDCl_3$) δ = 10.04 (d, *J* = 6.9 Hz, 1 H), 8.51 - 8.43 (m, 2 H), 7.95 - 7.85 (m, 3 H), 7.59 - 7.48 (m, 6 H), 7.45 - 7.40 (m, 1 H), 7.14 (t, *J* = 6.9 Hz, 1 H); **^{13}C NMR (125 MHz, $CDCl_3$)** δ = 153.6, 150.3, 145.7, 132.8, 132.0, 129.9, 129.5, 129.4, 129.3, 129.1, 128.4, 122.0, 117.2, 115.3

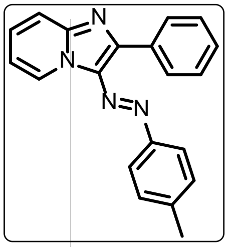

(*E*)-2-Phenyl-3-(*p*-tolyldiazenyl)imidazo[1,2-*a*]pyridine:

^{1}H NMR (500 MHz, $CDCl_3$) δ = 10.05 (d, *J* = 6.9 Hz, 1 H), 8.51 - 8.45 (m, 2 H), 7.91 (br. s., 1 H), 7.83 (d, *J* = 8.2 Hz, 2 H), 7.60 - 7.55 (m, 3 H), 7.53 - 7.48 (m, 1 H), 7.35 (d, *J* = 8.1 Hz, 2 H), 7.15 (t, *J* = 6.7 Hz, 1 H), 2.47 (s, 3 H); **^{13}C NMR (125 MHz, $CDCl_3$)** δ = 151.8, 149.4, 145.4, 140.0, 132.7, 132.0, 130.0, 129.9, 129.5, 129.4, 129.3, 128.5, 122.0, 117.2, 115.3, 21.4

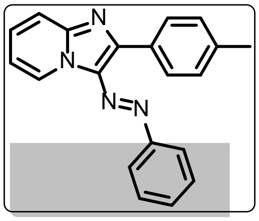

(*E*)-3-(Phenyldiazenyl)-2-(*p*-tolyl)imidazo[1,2-*a*]pyridine:

^{1}H NMR (500 MHz, $CDCl_3$) δ = 10.03 - 9.98 (m, 1 H), 8.36 (d, *J* = 8.2 Hz, 2 H), 7.89 - 7.87 (m, 2 H), 7.78 (d, *J* = 8.8 Hz, 1 H), 7.54 - 7.47 (m, 3 H), 7.42 - 7.38 (m, 1 H), 7.36 (d, *J* = 7.9 Hz, 2 H), 7.08 - 7.05 (m, 1 H), 2.46 (s, 3 H); **^{13}C NMR (125 MHz, $CDCl_3$)** δ = 153.8, 150.7, 145.9, 139.5, 131.9, 130.1, 129.9, 129.5, 129.4, 129.3, 129.1, 122.0, 117.2, 115.2, 21.4

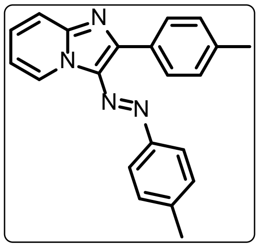

(*E*)-2-(*p*-Tolyl)-3-(*p*-tolyldiazenyl)imidazo[1,2-*a*]pyridine:

^{1}H NMR (500 MHz, $CDCl_3$) δ = 10.04 (d, *J* = 6.9 Hz, 1 H), 8.37 (d, *J* = 8.1 Hz, 2 H), 7.95 (d like, *J* = 7.6 Hz, 1 H), 7.80 (d, *J* = 8.2 Hz, 2 H), 7.57 (t, *J* = 7.8 Hz, 1 H), 7.37 (d, *J* = 7.9 Hz, 2 H), 7.33 (d, *J* = 8.1 Hz, 2 H), 7.14 (t, *J* = 6.8 Hz, 1 H), 2.46 (d like, *J* = 2.7 Hz, 6 H); **^{13}C NMR (125 MHz, $CDCl_3$)** δ = 151.8, 150.1, 145.7, 139.6, 139.2, 131.8, 130.3, 129.8, 129.7, 129.3, 129.2, 129.1, 121.9, 117.2, 114.9, 21.4, 21.3

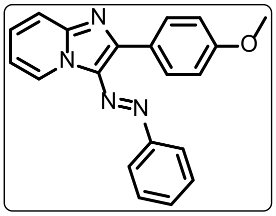

(*E*)-2-(4-Methoxyphenyl)-3-(phenyldiazenyl)imidazo[1,2-*a*]pyridine:

^{1}H NMR (500 MHz, $CDCl_3$) δ = 10.07 - 10.01 (m, 1 H), 8.49 - 8.42 (m, 2 H), 7.91 - 7.86 (m, 2 H), 7.84 (d, *J* = 8.8 Hz, 1 H), 7.57 - 7.50 (m, 3 H), 7.43 - 7.38 (m, 1 H), 7.13 - 7.06 (m, 3 H), 3.92 (s, 3 H); **^{13}C NMR (125 MHz, $CDCl_3$)** δ = 160.8, 153.8, 150.6, 146.0, 131.7, 131.3, 129.4, 129.3, 129.1, 125.6, 121.9, 117.0, 114.9, 113.9, 55.3

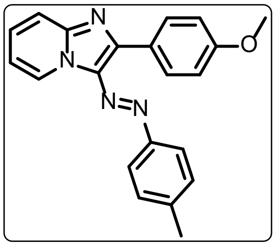

(*E*)-2-(4-Methoxyphenyl)-3-(*p*-tolyldiazenyl)imidazo[1,2-*a*]pyridine:

^{1}H NMR (500 MHz, CDCl$_3$) δ = 10.03 (d, *J* = 6.9 Hz, 1 H), 8.48 - 8.41 (m, 2 H), 7.86 (d like, *J* = 8.5 Hz, 1 H), 7.79 (d, *J* = 8.2 Hz, 2 H), 7.53 (t, *J* = 7.9 Hz, 1 H), 7.33 (d, *J* = 7.9 Hz, 2 H), 7.12 - 7.05 (m, 3 H), 3.92 (s, 3 H), 2.45 (s, 3 H); **^{13}C NMR (125 MHz, CDCl$_3$)** δ = 160.6, 151.7, 149.8, 145.7, 139.3, 131.5, 131.2, 129.6, 129.2, 129.0, 125.7, 121.7, 116.9, 114.6, 113.8, 55.2, 21.3

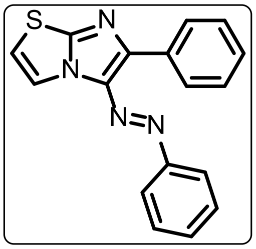

(*E*)-6-Phenyl-5-(phenyldiazenyl)imidazo[2,1-*b*]thiazole:

^{1}H NMR (500 MHz, CDCl$_3$) δ = 8.59 (d, *J* = 4.4 Hz, 1 H), 8.41 - 8.37 (m, 2 H), 7.89 - 7.87 (m, 2 H), 7.54 - 7.50 (m, 4 H), 7.46 - 7.40 (m, 2 H), 6.99 (d, *J* = 4.4 Hz, 1 H); **^{13}C NMR (125 MHz, CDCl$_3$)** δ = 154.2, 153.2, 149.9, 135.8, 133.0, 129.7, 129.1, 129.0, 128.9, 128.5, 123.1, 122.2, 113.3

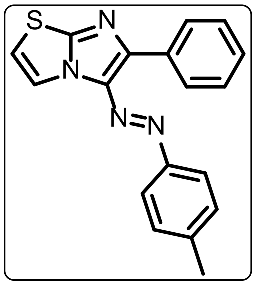

(*E*)-6-Phenyl-5-(*p*-tolyldiazenyl)imidazo[2,1-*b*]thiazole:

^{1}H NMR (500 MHz, CDCl$_3$) δ = 8.58 - 8.53 (m, 1 H), 8.43 - 8.35 (m, 2 H), 7.82 - 7.74 (m, 2 H), 7.51 (t, *J* = 7.7 Hz, 2 H), 7.43 (t, *J* = 7.3 Hz, 1 H), 7.31 (d, *J* = 7.9 Hz, 2 H), 6.93 (t, *J* = 4.4 Hz, 1 H), 2.45 (s, 3 H) **^{13}C NMR (125 MHz, CDCl$_3$)** δ = 153.9, 151.4, 149.3, 140.2, 135.9, 133.2, 129.8, 128.9, 128.8, 128.5, 123.1, 122.2, 113.1, 21.4

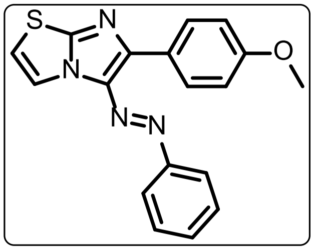

(*E*)-6-(4-Methoxyphenyl)-5-(phenyldiazenyl)imidazo[2,1-*b*]thiazole:

^{1}H NMR (500 MHz, CDCl$_3$) δ = 8.53 (dd, *J* = 1.8, 4.3 Hz, 1 H), 8.35 (d, *J* = 8.9 Hz, 2 H), 7.84 (d, *J* = 8.1 Hz, 2 H), 7.50 (t, *J* = 7.7 Hz, 2 H), 7.39 (t, *J* = 7.3 Hz, 1 H), 7.04 (d, *J* = 8.9 Hz, 2 H), 6.89 (dd, *J* = 1.4, 4.4 Hz, 1 H), 3.89 (s, 3 H); **^{13}C NMR (125 MHz, CDCl$_3$)** δ = 160.5, 154.5, 153.4, 150.3, 135.4, 130.4, 129.4, 129.1, 125.9, 123.2, 122.1, 114.1, 112.7, 55.3

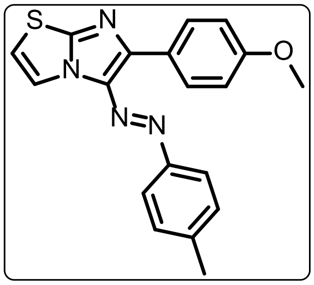

(*E*)-6-(4-Methoxyphenyl)-5-(*p*-tolyldiazenyl)imidazo[2,1-*b*]thiazole:

^{1}H NMR (500 MHz, CDCl$_3$) δ = 8.54 - 8.47 (m, 1 H), 8.34 (d, *J* = 8.8 Hz, 2 H), 7.73 (d, *J* = 8.1 Hz, 2 H), 7.31 - 7.26 (m, 2 H), 7.03 (d, *J* = 8.8 Hz, 2 H), 6.90 - 6.82 (m, 1 H), 3.89 (s, 3 H), 2.43 (s, 3 H); **^{13}C NMR (125 MHz, CDCl$_3$)** δ = 160.4, 154.1, 151.4, 149.6, 139.8, 135.4, 130.3, 129.7, 126.0, 123.2, 122.0, 114.0, 112.5, 55.3, 21.4

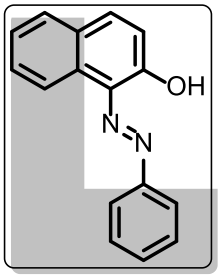

(*E*)-1-(Phenyldiazenyl)naphthalen-2-ol:

^{1}H NMR (200 MHz, $CDCl_3$) δ = 8.54 (d, *J* = 8.2 Hz, 1 H), 7.76 - 7.65 (m, 3 H), 7.60 - 7.29 (m, 6 H), 6.85 (d, *J* = 9.4 Hz, 1 H); **^{13}C NMR (50 MHz, $CDCl_3$)** δ = 172.0, 144.7, 140.1, 133.6, 130.0, 129.6, 128.8, 128.6, 128.0, 127.4, 125.7, 124.8, 121.7, 118.5

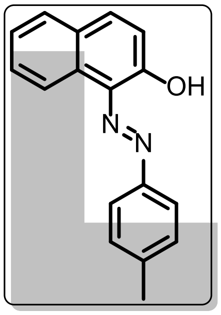

(*E*)-1-(*p*-Tolyldiazenyl)naphthalen-2-ol:

^{1}H NMR (500 MHz, $CDCl_3$) δ = 8.62 (d, *J* = 8.4 Hz, 1 H), 7.75 (d, *J* = 9.3 Hz, 1 H), 7.71 - 7.67 (m, 2 H), 7.64 (d, *J* = 7.9 Hz, 1 H), 7.57 (ddd, *J* = 1.3, 7.1, 8.3 Hz, 1 H), 7.41 (ddd, *J* = 1.2, 7.0, 7.9 Hz, 1 H), 7.30 (d, *J* = 7.9 Hz, 2 H), 6.96 (d, *J* = 9.3 Hz, 1 H), 2.43 (s, 3 H); **^{13}C NMR (125 MHz, $CDCl_3$)** δ = 168.3, 143.5, 138.8, 138.3, 133.5, 130.1, 129.7, 128.5, 128.4, 128.0, 125.3, 123.9, 121.6, 119.1, 21.2

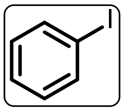

Iodobenzene:

^{1}H NMR (500MHz, $CDCl_3$) δ = 7.73 (d, *J* = 8.1 Hz, 2 H), 7.35 (dt, *J* = 0.8, 7.5 Hz, 1 H), 7.16 - 7.09 (m, 2 H); **^{13}C NMR (125 MHz, $CDCl_3$)** δ = 137.3, 130.1, 127.3, 94.4

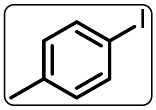

1-Iodo-4-methylbenzene:

^{1}H NMR (500MHz, $CDCl_3$) δ = 7.57 (d, *J* = 8.2 Hz, 2 H), 6.94 (d, *J* = 7.8 Hz, 2 H), 2.30 (s, 3 H); **^{13}C NMR (125 MHz, $CDCl_3$)** δ = 137.4, 137.2, 131.1, 90.2, 21.0

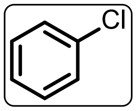

Chlorobenzene:

^{1}H NMR (500MHz, $CDCl_3$) δ = 7.29 - 7.25 (m, 2 H), 7.24 - 7.20 (m, 2 H), 7.19 - 7.15 (m, 1 H); **^{13}C NMR (126MHz, $CDCl_3$)** δ = 134.2, 129.7, 128.6, 126.4

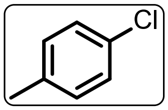

1-Chloro-4-methylbenzene:

^{1}H NMR (500 MHz, $CDCl_3$) δ = 7.28 (d, *J* = 8.2 Hz, 2 H), 7.16 (d, *J* = 8.1 Hz, 2 H), 2.38 (s, 3 H); **^{13}C NMR (125 MHz, $CDCl_3$)** δ = 136.2, 131.1, 130.3, 128.2, 20.8

2-Phenylfuran:

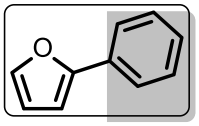

^{1}H NMR (500 MHz, $CDCl_3$) δ = 7.74 - 7.66 (m, 2 H), 7.52 - 7.45 (m, 1 H), 7.40 (t, *J* = 7.7 Hz, 2 H), 7.30 - 7.25 (m, 1 H), 6.67 (d, *J* = 3.4 Hz, 1 H), 6.49 (dd, *J* = 1.8, 3.4 Hz, 1 H); **^{13}C NMR (125 MHz, $CDCl_3$)** δ = 154.0, 142.0, 130.9, 128.6, 127.3, 123.8, 111.6, 104.9

2-(*p*-Tolyl)furan:

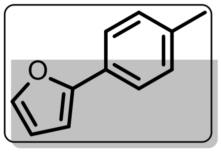

^{1}H NMR (500 MHz, $CDCl_3$) δ = 7.60 (d, *J* = 8.2 Hz, 2 H), 7.47 (s, 1 H), 7.22 (d, *J* = 7.9 Hz, 2 H), 6.62 (d, *J* = 3.2 Hz, 1 H), 6.48 (dd, *J* = 1.6, 3.1 Hz, 1 H), 2.39 (s, 3 H); **^{13}C NMR (125 MHz, $CDCl_3$)** δ = 154.2, 141.6, 137.1, 129.3, 128.2, 123.7, 111.5, 104.2, 21.1

Phenyl(*p*-tolyl)sulfane:

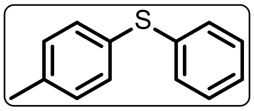

^{1}H NMR (500 MHz, $CDCl_3$) δ = 7.32 (d, *J* = 8.2 Hz, 2 H), 7.29 - 7.26 (m, 4 H), 7.23 - 7.18 (m, 1 H), 7.15 (d, *J* = 7.9 Hz, 2 H), 2.36 (s, 3 H); **^{13}C NMR (125 MHz, $CDCl_3$)** δ = 137.5, 137.1, 132.2, 131.3, 130.0, 129.8, 129.0, 126.4, 21.1

Di-*p*-tolylsulfane:

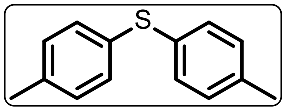

^{1}H NMR (500 MHz, $CDCl_3$) δ = 7.25 (d, *J* = 8.1 Hz, 4 H), 7.12 (d, *J* = 7.9 Hz, 4 H), 2.34 (s, 6 H); **^{13}C NMR (125 MHz, $CDCl_3$)** δ = 136.8, 132.7, 131.0, 129.8, 21.0

Azidobenzene:

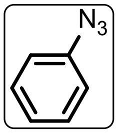

^{1}H NMR (500 MHz, $CDCl_3$) δ = 7.39 - 7.34 (m, 2 H), 7.18 - 7.13 (m, 1 H), 7.07 - 7.02 (m, 2 H); **^{13}C NMR (75 MHz, $CDCl_3$)** δ = 139.9, 129.7, 124.8, 119.0

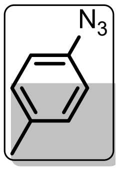

1-Azido-4-methylbenzene:

^{1}H NMR (500 MHz, $CDCl_3$) δ = 7.16 (d, *J* = 7.9 Hz, 2 H), 6.93 (d, *J* = 8.4 Hz, 2 H), 2.34 (s, 3 H); **^{13}C NMR (75 MHz, $CDCl_3$)** δ = 137.1, 134.5, 130.3, 118.8, 20.7

9) Supplementary References

1. Ahn, G. N. *et al.* A numbering-up metal microreactor for the high-Throughput production of a commercial drug by copper catalysis. *Lab Chip* **19**, 3535–3542 (2019).

2. Hari, D. P., Schroll, P. & König, B. Metal-free, visible-light-mediated direct C-H arylation of heteroarenes with aryl diazonium salts. *J. Am. Chem. Soc.* **134**, 2958–2961 (2012).

3. Baig, M. F. *et al.* Synthesis and biological evaluation of imidazo[2,1-b]thiazole-benzimidazole conjugates as microtubule-targeting agents. *Bioorg. Chem.* **77**, 515–526 (2018).

4. Shevlin, M. Practical High-Throughput Experimentation for Chemists. *ACS Med. Chem. Lett.* **8**, 601–607 (2017).

5. Santanilla, A. B. *et al.* Nanomole-scale high-throughput chemistry for the synthesis of complex molecules. *Science (80-.).* **347**, 443–448 (2015).

6. Krska, S. W., DiRocco, D. A., Dreher, S. D. & Shevlin, M. The Evolution of Chemical High-Throughput Experimentation to Address Challenging Problems in Pharmaceutical Synthesis. *Acc. Chem. Res.* **50**, 2976–2985 (2017).

7. Mennen, S. M. *et al.* The Evolution of High-Throughput Experimentation in Pharmaceutical Development and Perspectives on the Future. *Org. Process Res. Dev.* **23**, 1213–1242 (2019).

8. Welch, C. J. High throughput analysis enables high throughput experimentation in pharmaceutical process research. *React. Chem. Eng.* **4**, 1895–1911 (2019).

9. Hwang, Y. J. *et al.* A segmented flow platform for on-demand medicinal chemistry and compound synthesis in oscillating droplets. *Chem. Commun.* **53**, 6649–6652 (2017).

10. Epps, R. W. *et al.* Artificial Chemist: An Autonomous Quantum Dot Synthesis Bot. *Adv. Mater.* **32**, 1–9 (2020).

11. De Bellefon, C. *et al.* High-throughput screening of molecular catalysts using automated liquid handling, injection, and microdevices. *Chimia (Aarau).* **56**, 621–626 (2002).

12. Perera, D. *et al.* A platform for automated nanomole-scale reaction screening and micromole-scale synthesis in flow. *Science (80-.).* **359**, 429–434 (2018).

13. Wang, M. *et al.* Enzyme Degassing for Oxygen-Sensitive Reactions in Open Vessels of an Automated Parallel Synthesizer: RAFT Polymerizations. *ACS Comb. Sci.* **21**, 643–649 (2019).

14. Guerrero-Sanchez, C. *et al.* Quasi-block copolymer libraries on demand via sequential RAFT polymerization in an automated parallel synthesizer. *Polym. Chem.* **4**, 1857–1862 (2013).

15. De Bellefon, C., Tanchoux, N., Caravieilhes, S., Grenouillet, P. & Hessel, V. Microreactors for dynamic, high throughput screening of fluid/liquid molecular catalysis. *Angew. Chemie (International Ed. English)* **39**, 3442–3445 (2000).

16. Chatterjee, S., Guidi, M., Seeberger, P. H. & Gilmore, K. Automated radial synthesis of organic molecules. *Nature* **579**, 379–384 (2020).

10) NMR copies of all synthesized compounds (3a-3x)

Supplementary Figure 9: ^{1}H-NMR spectra of (*E*)-2-Phenyl-3-(phenyldiazenyl)imidazo[1,2-*a*]pyridine

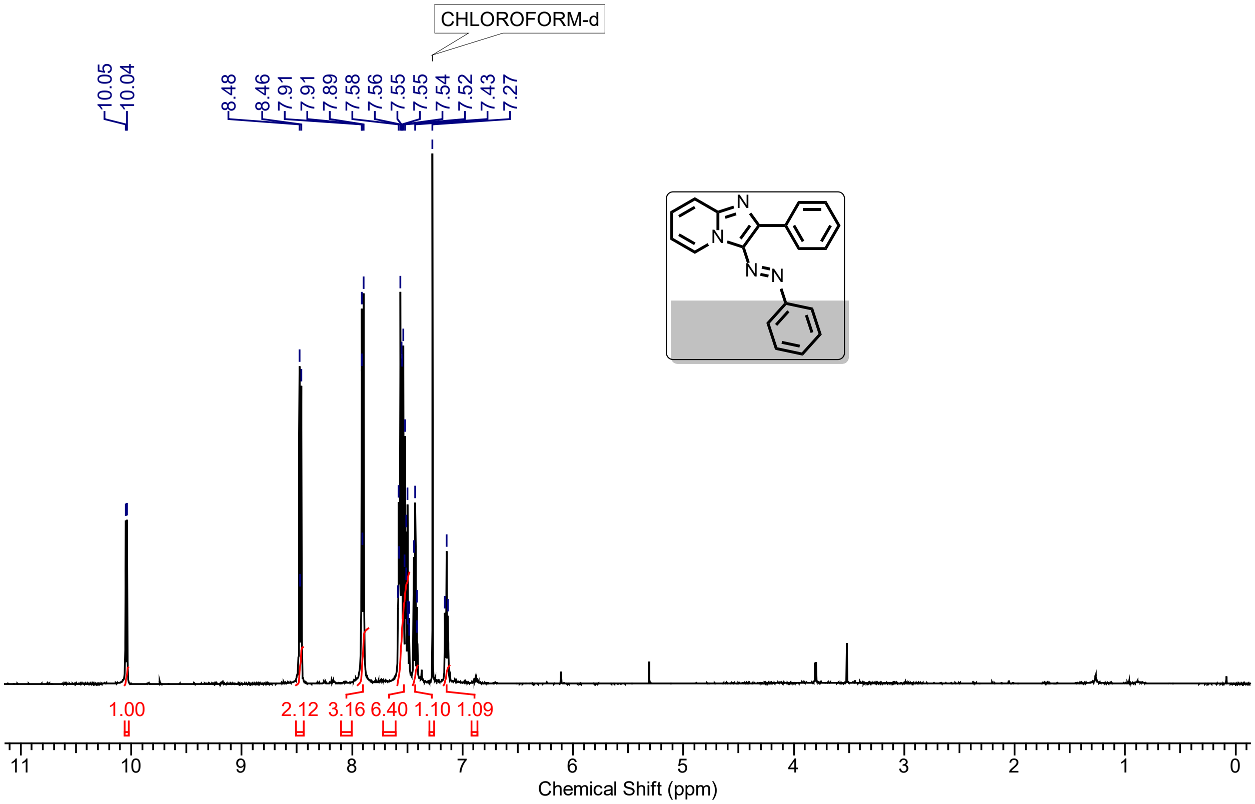

Supplementary Figure 10: ^{13}C-NMR spectra of (*E*)-2-Phenyl-3-(phenyldiazenyl)imidazo[1,2-*a*]pyridine

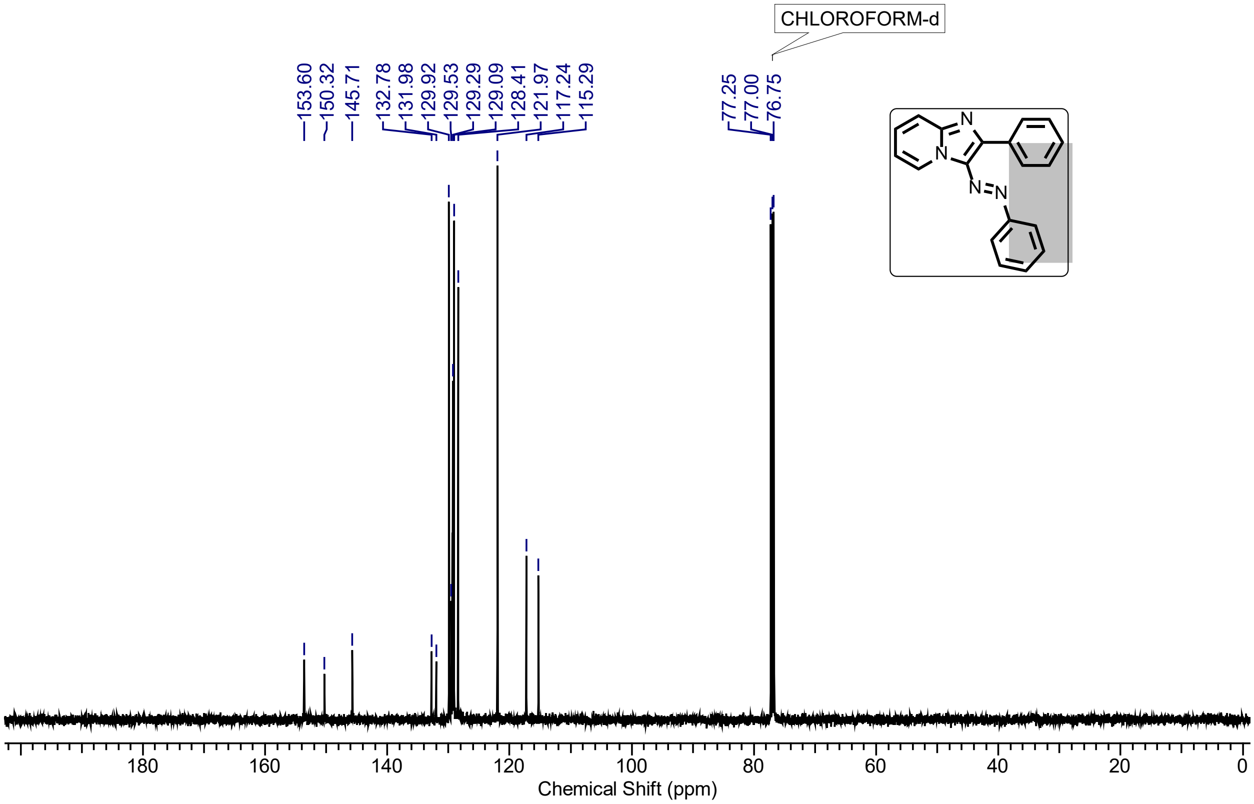

Supplementary Figure 11: ^{1}H-NMR spectra of (E)-2-Phenyl-3-(p-tolyldiazenyl)imidazo[1,2-a]pyridine

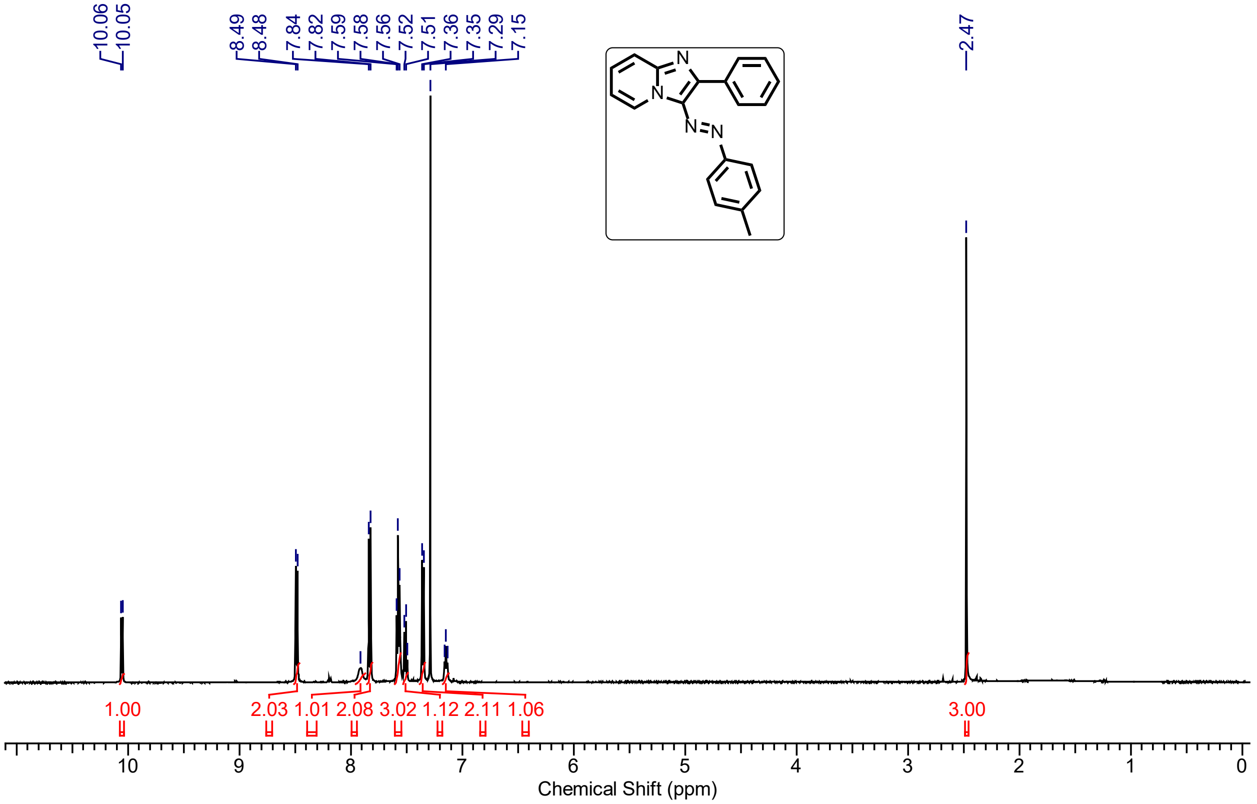

Supplementary Figure 12: ^{13}C-NMR spectra of (E)-2-Phenyl-3-(p-tolyldiazenyl)imidazo[1,2-a]pyridine

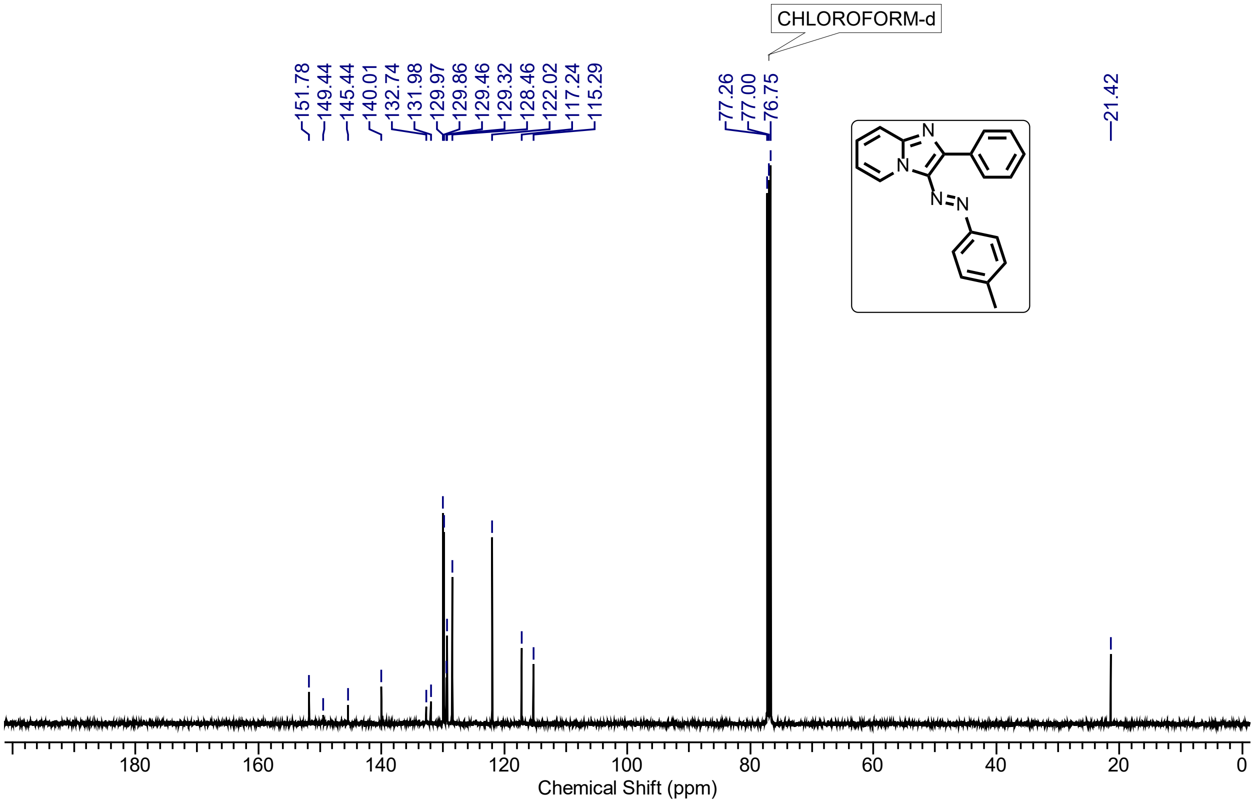

Supplementary Figure 13: ^{1}H-NMR spectra of (E)-3-(Phenyldiazenyl)-2-(p-tolyl)imidazo[1,2-a]pyridine

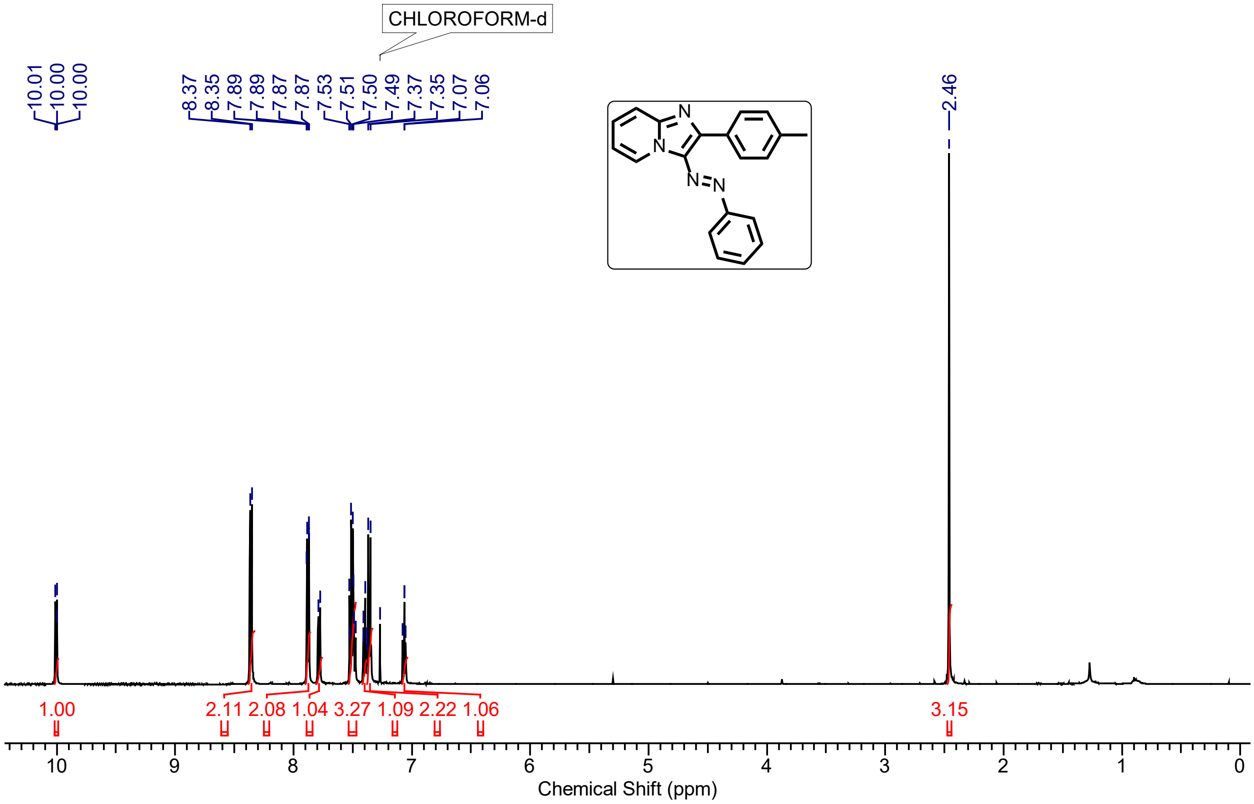

Supplementary Figure 14: ^{13}C-NMR spectra of (E)-3-(Phenyldiazenyl)-2-(p-tolyl)imidazo[1,2-a]pyridine

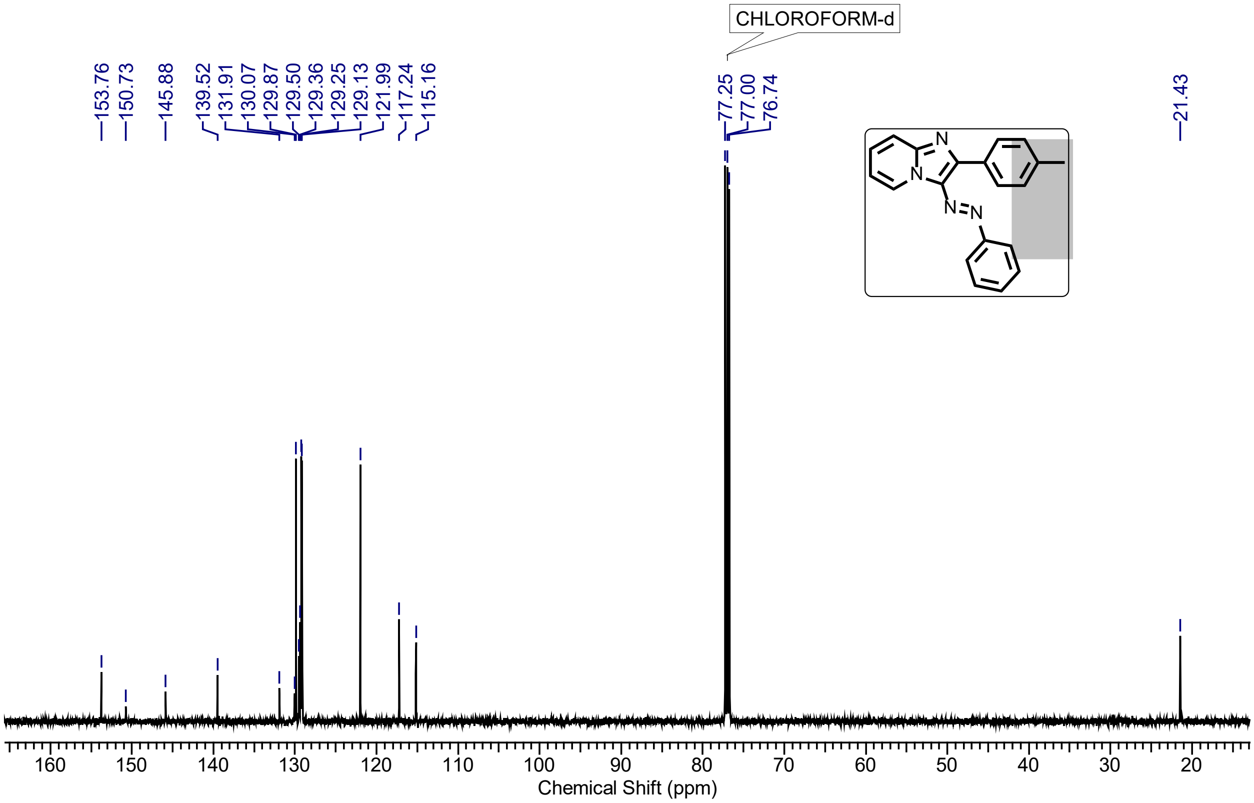

Supplementary Figure 15: ^{1}H-NMR spectra of (E)-2-(p-Tolyl)-3-(p-tolyldiazenyl)imidazo[1,2-a]pyridine

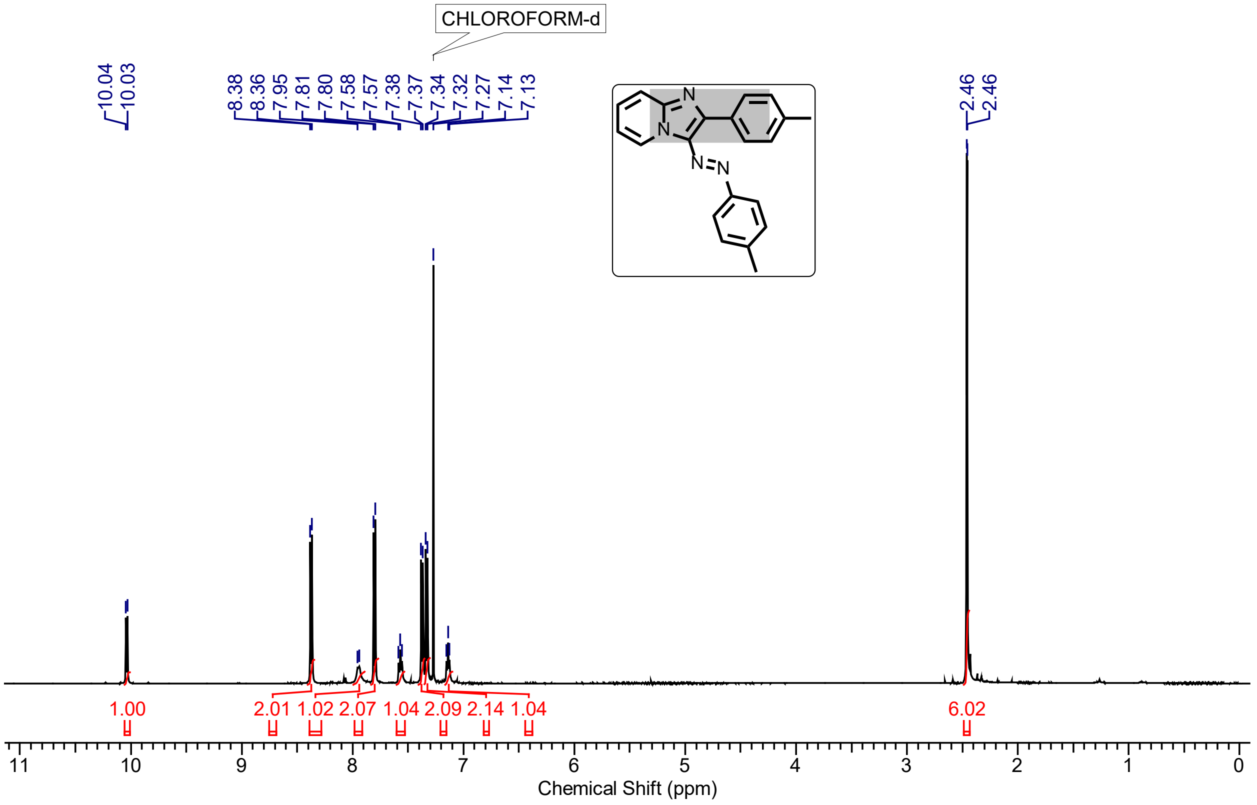

Supplementary Figure 16: ^{13}C-NMR spectra of (E)-2-(p-Tolyl)-3-(p-tolyldiazenyl)imidazo[1,2-a]pyridine

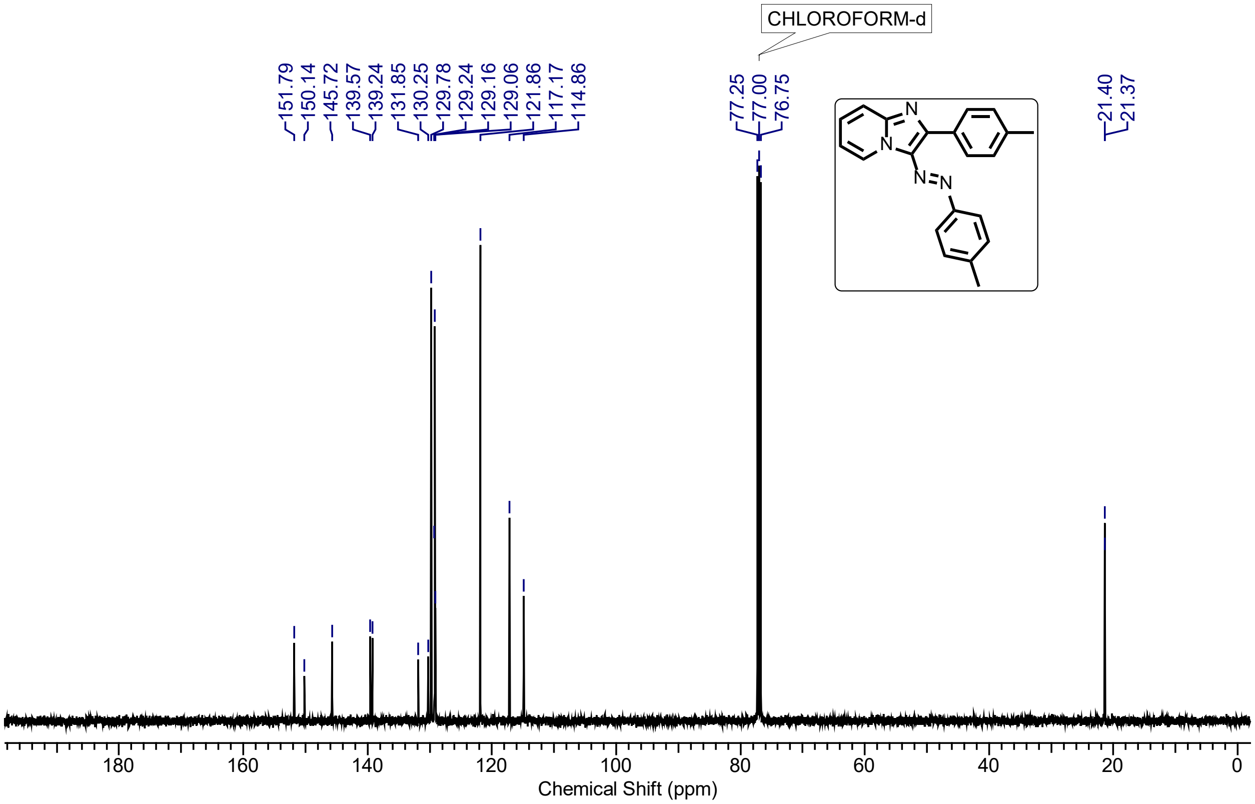

Supplementary Figure 17: ^{1}H-NMR spectra of (E)-2-(4-Methoxyphenyl)-3-(phenyldiazenyl)imidazo[1,2-a]pyridine

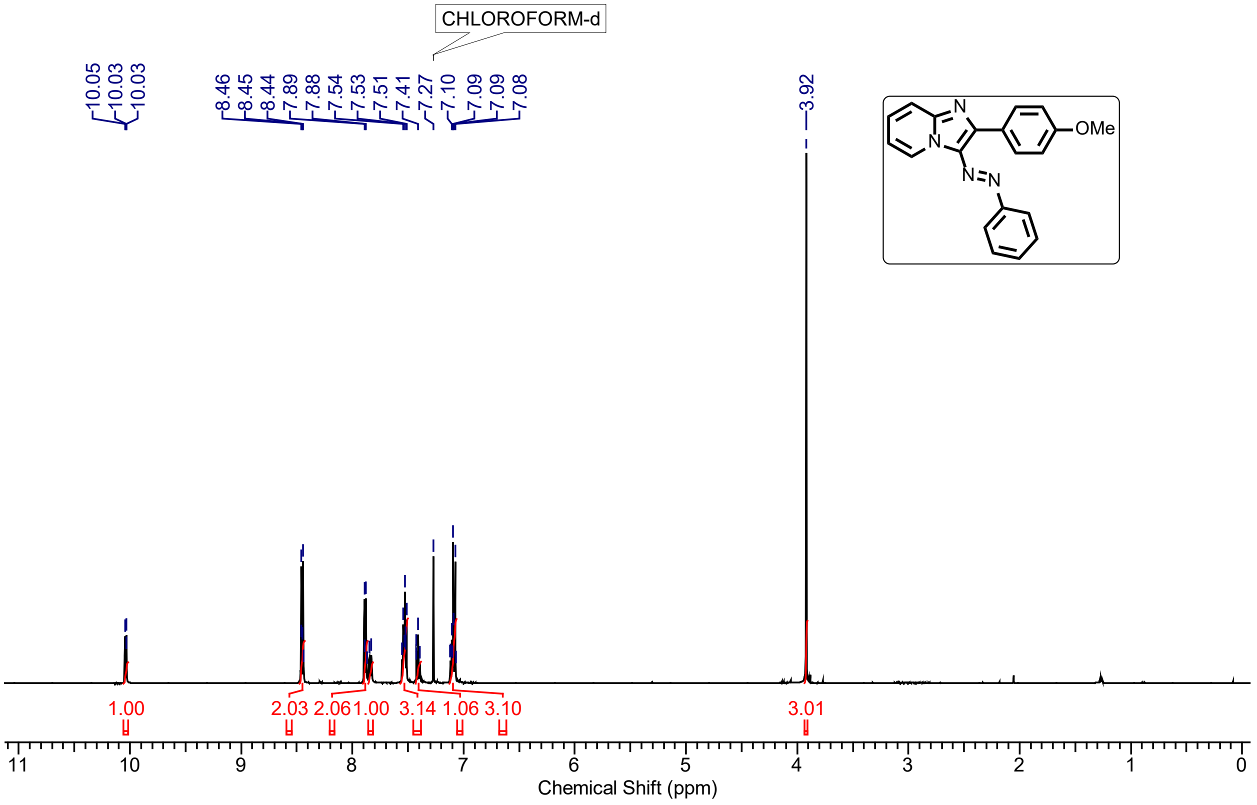

Supplementary Figure 18: ^{13}C-NMR spectra of (E)-2-(4-Methoxyphenyl)-3-(phenyldiazenyl)imidazo[1,2-a]pyridine

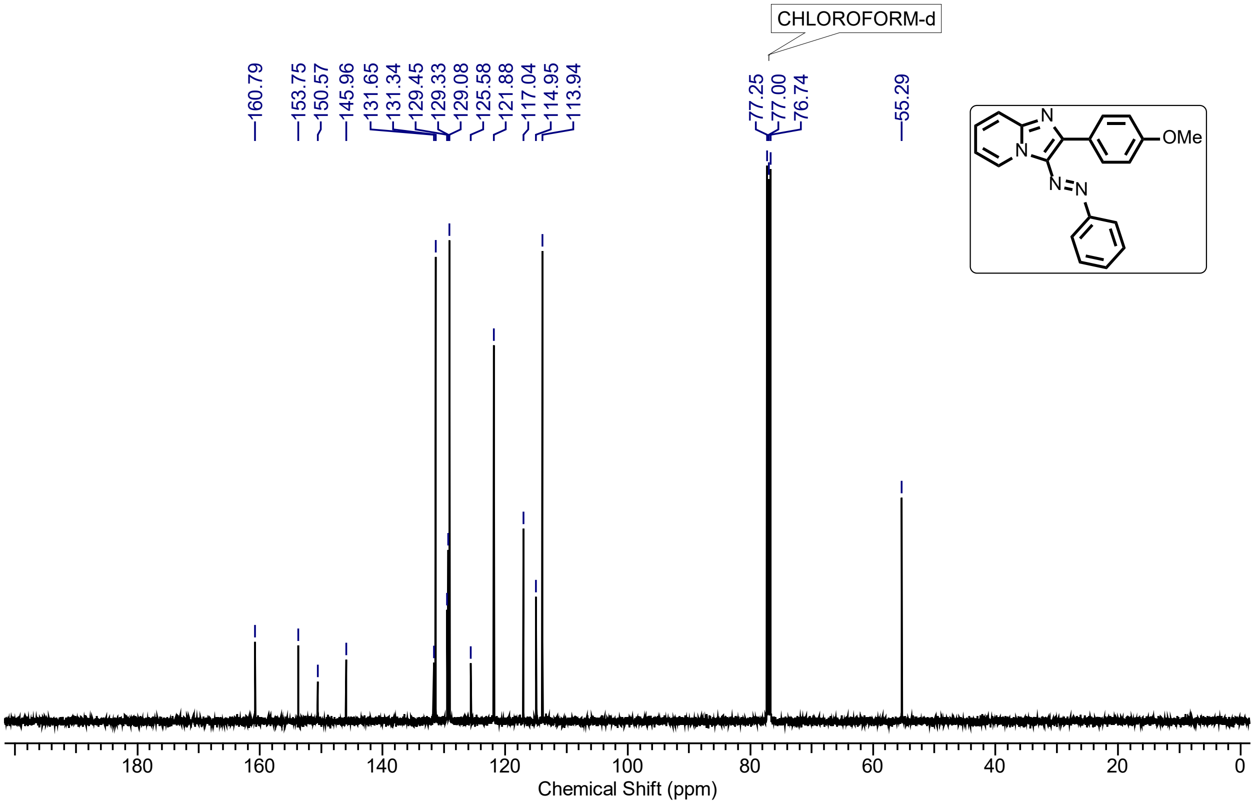

Supplementary Figure 19: ^{1}H-NMR spectra of (E)-2-(4-Methoxyphenyl)-3-(p-tolyldiazenyl)imidazo[1,2-a]pyridine

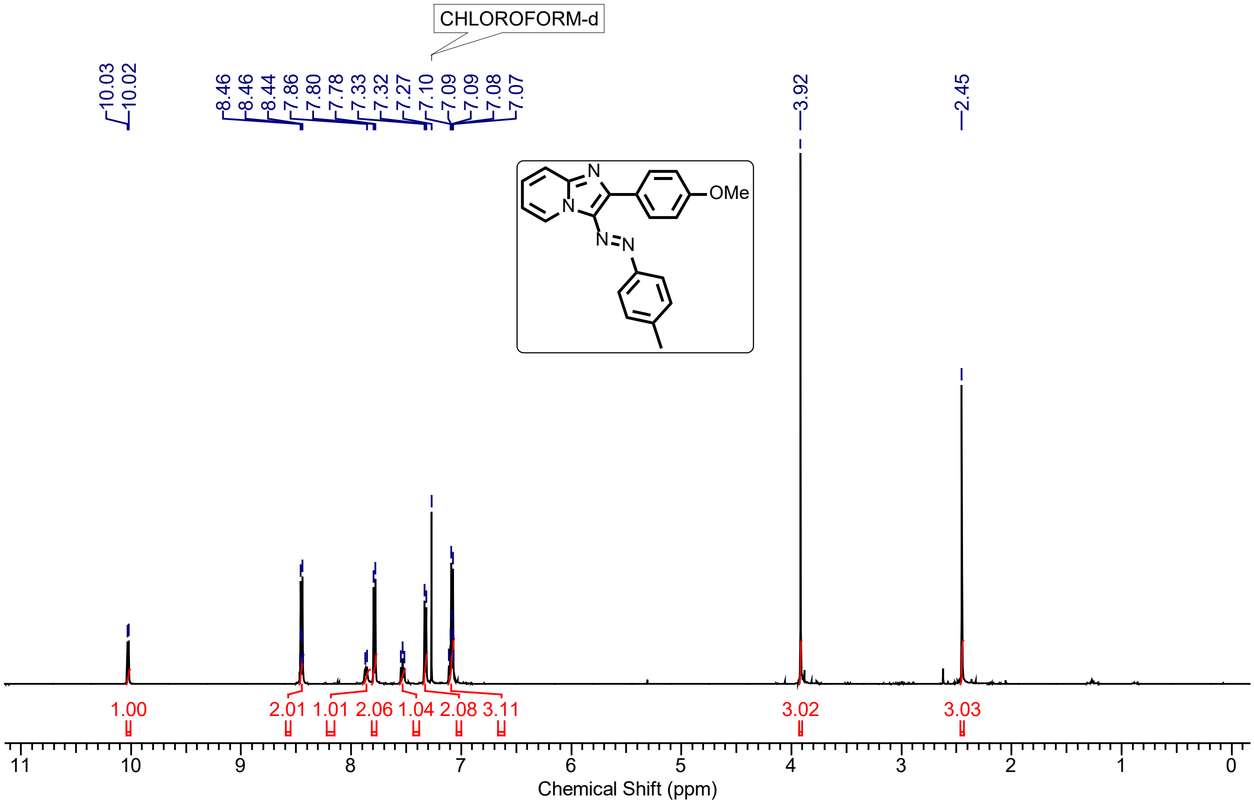

Supplementary Figure 20: ^{13}C-NMR spectra of (E)-2-(4-Methoxyphenyl)-3-(p-tolyldiazenyl)imidazo[1,2-a]pyridine

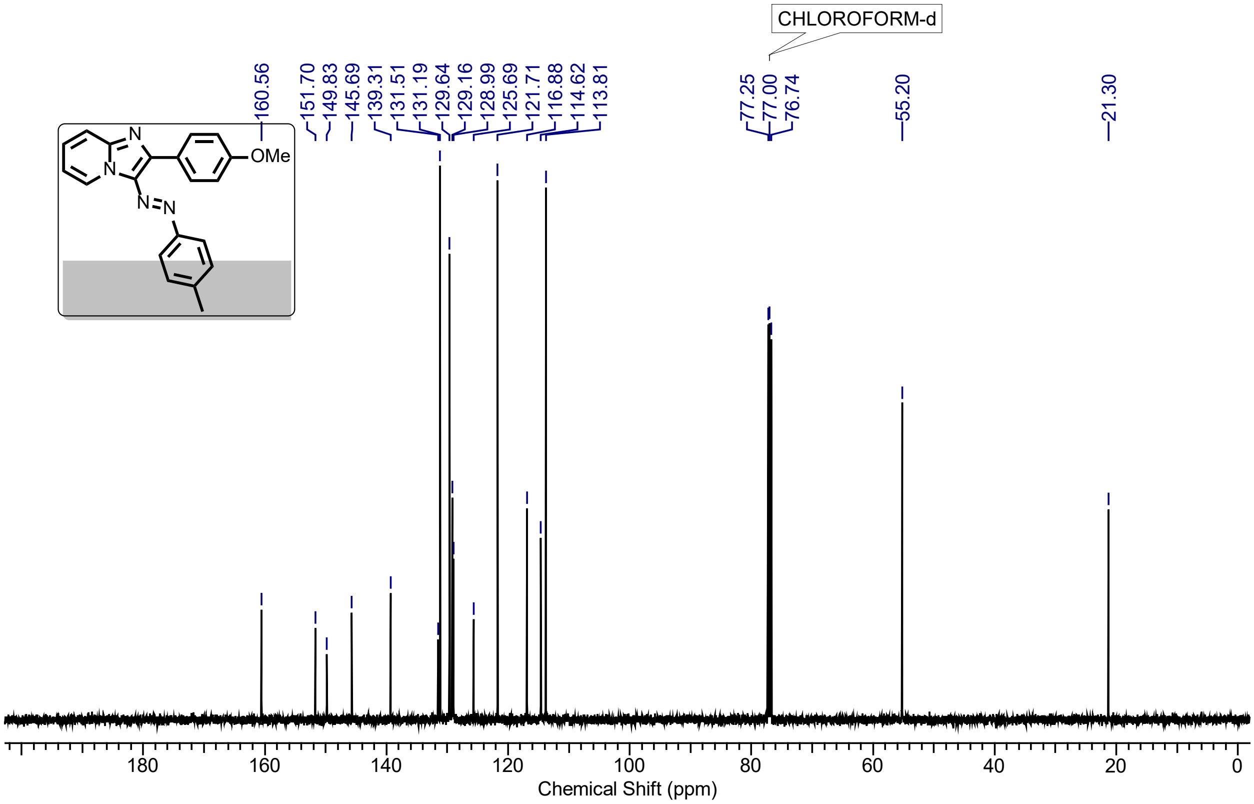

Supplementary Figure 21: ^{1}H-NMR spectra of (E)-6-Phenyl-5-(phenyldiazenyl)imidazo[2,1-b]thiazole

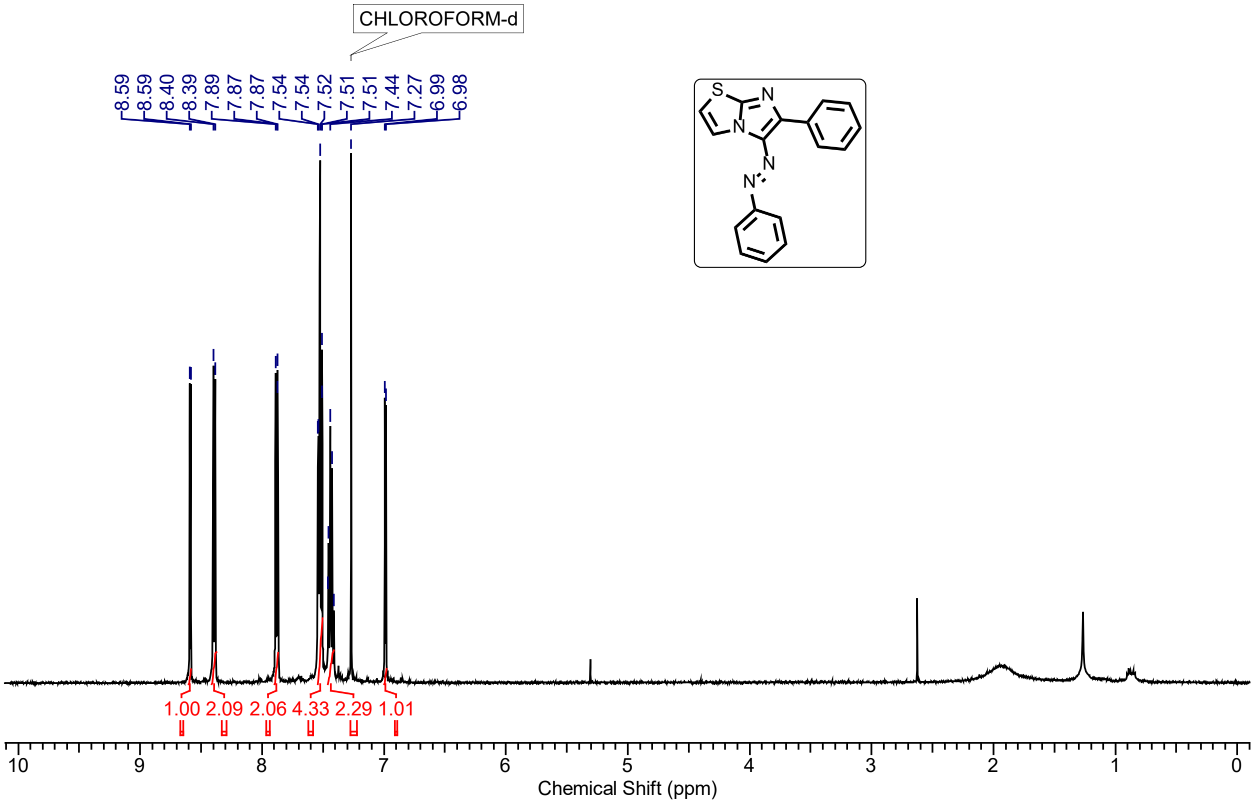

Supplementary Figure 22: ^{13}C-NMR spectra of (E)-6-Phenyl-5-(phenyldiazenyl)imidazo[2,1-b]thiazole

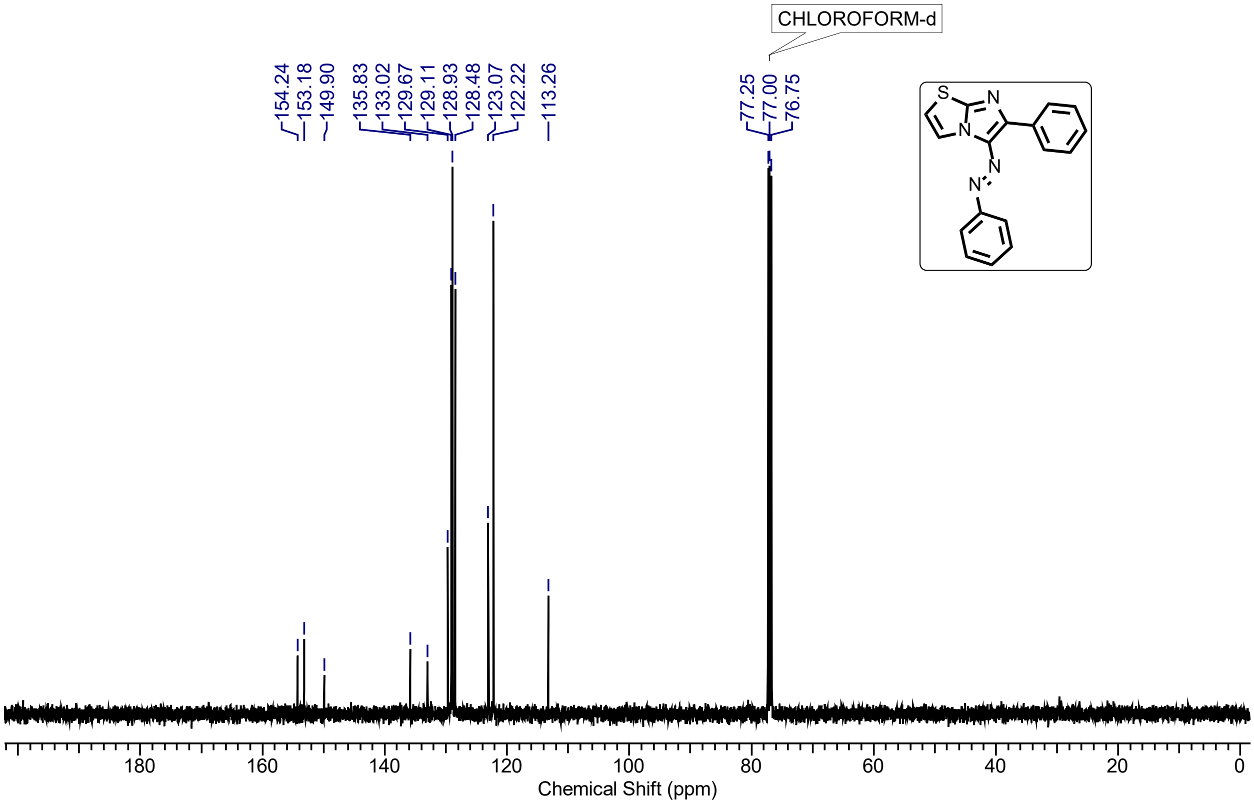

Supplementary Figure 23: ^{1}H-NMR spectra of (E)-6-Phenyl-5-(p-tolyldiazenyl)imidazo[2,1-b]thiazole

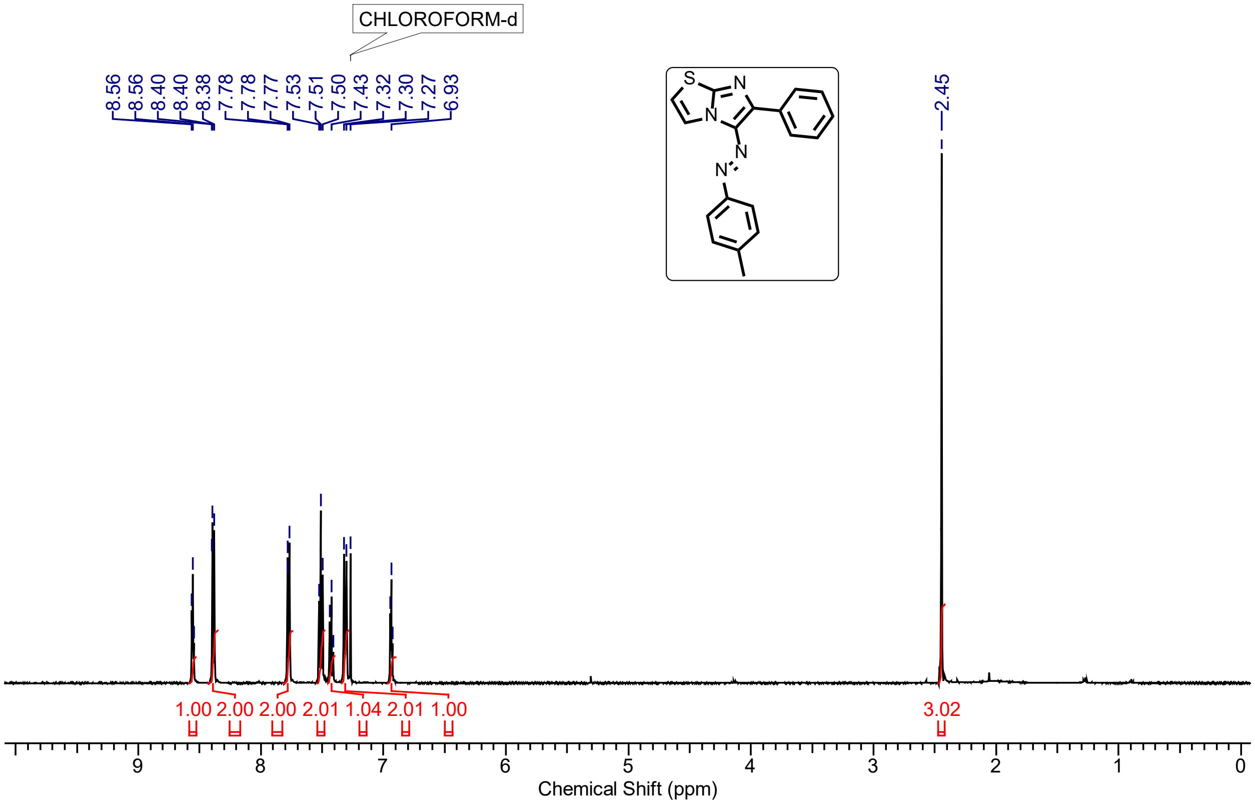

Supplementary Figure 24: ^{13}C-NMR spectra of (E)-6-Phenyl-5-(p-tolyldiazenyl)imidazo[2,1-b]thiazole

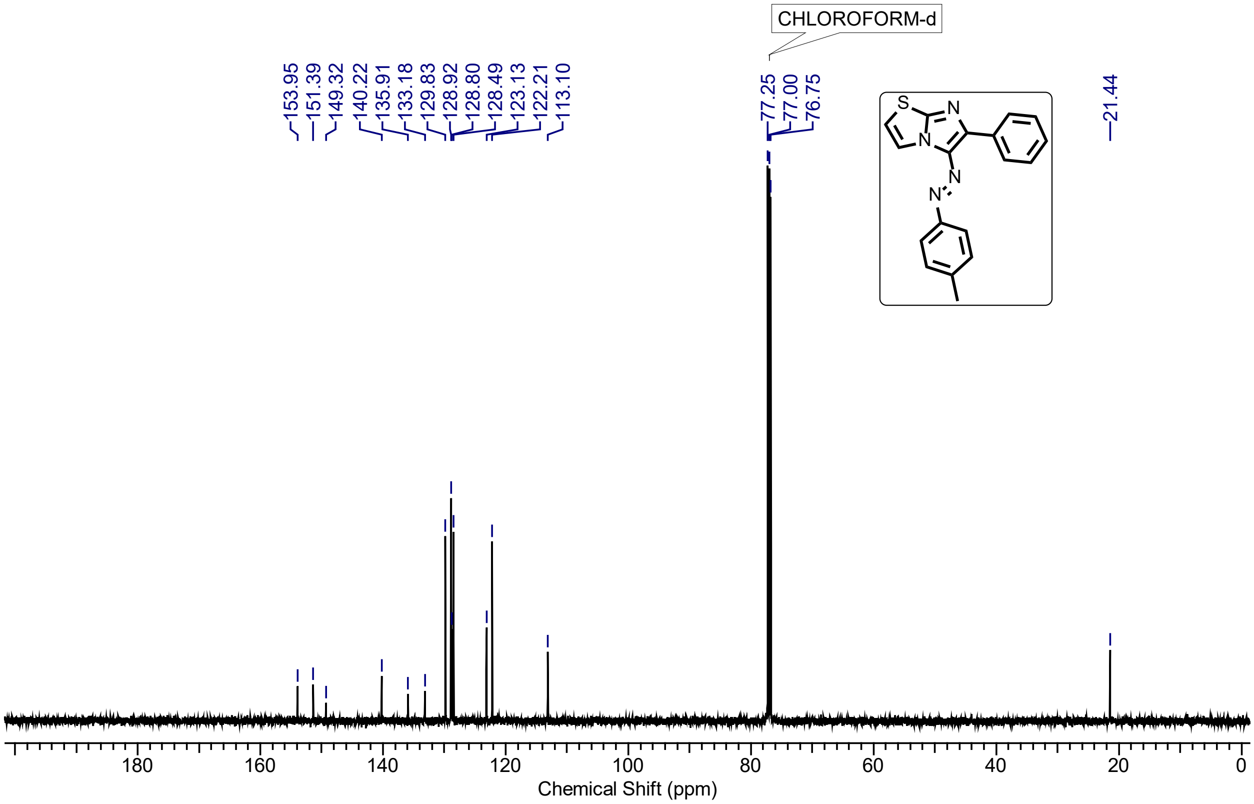

Supplementary Figure 25: ^{1}H-NMR spectra of (E)-6-(4-Methoxyphenyl)-5-(phenyldiazenyl)imidazo[2,1-b]thiazole

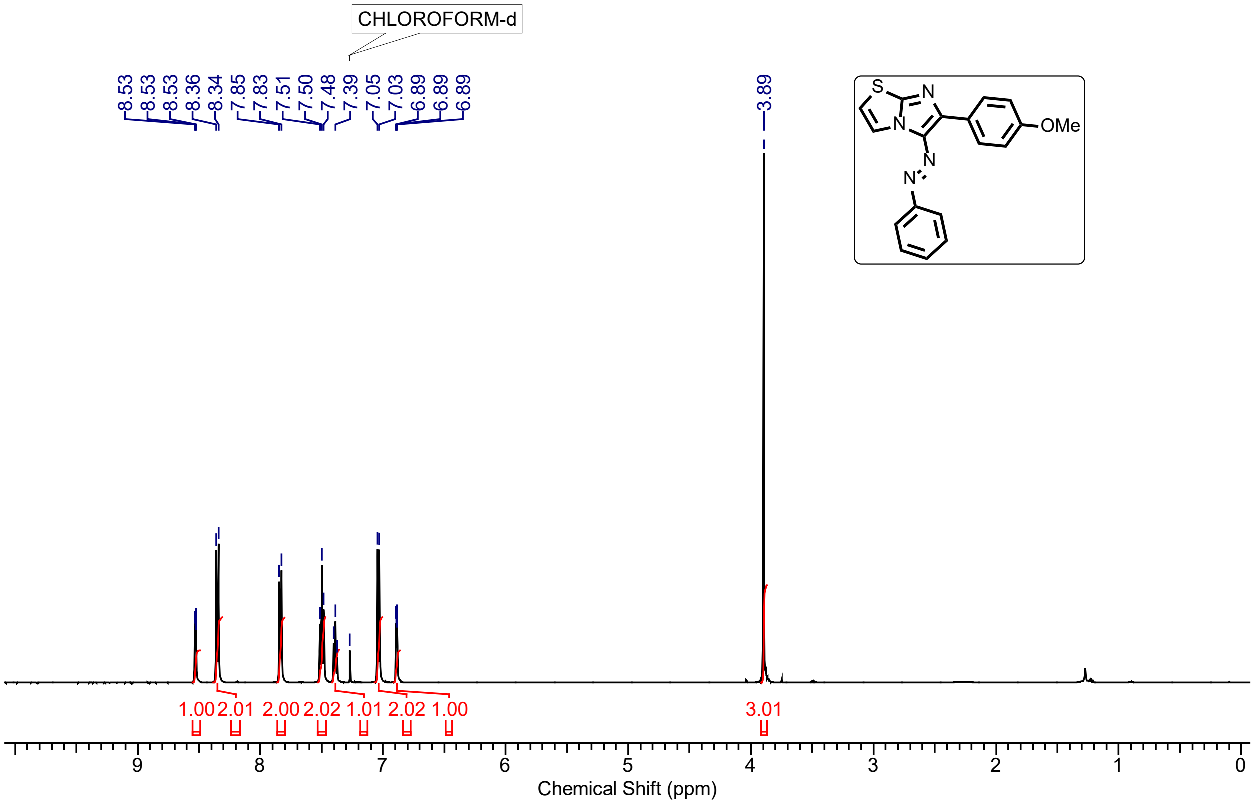

Supplementary Figure 26: ^{13}C-NMR spectra of (E)-6-(4-Methoxyphenyl)-5-(phenyldiazenyl)imidazo[2,1-b]thiazole

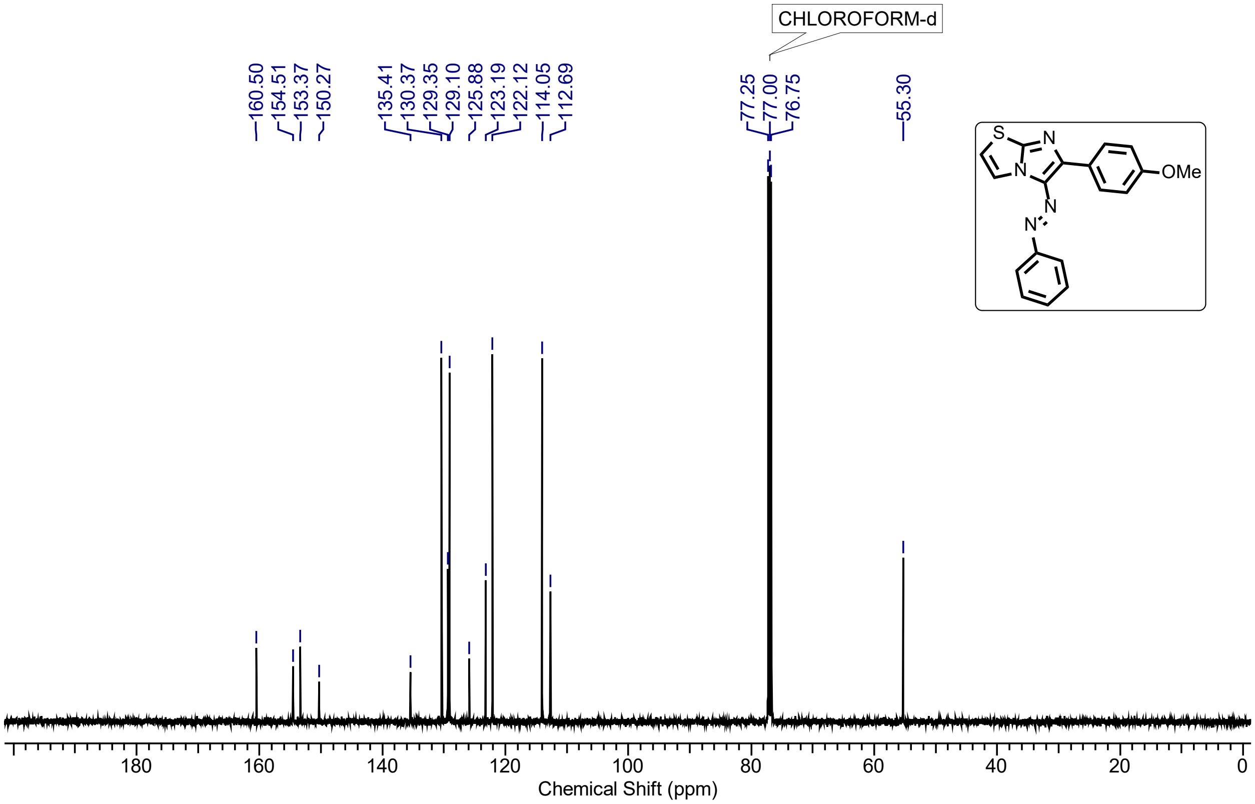

Supplementary Figure 27: ^{1}H-NMR spectra of (E)-6-(4-Methoxyphenyl)-5-(p-tolyldiazenyl)imidazo[2,1-b]thiazole

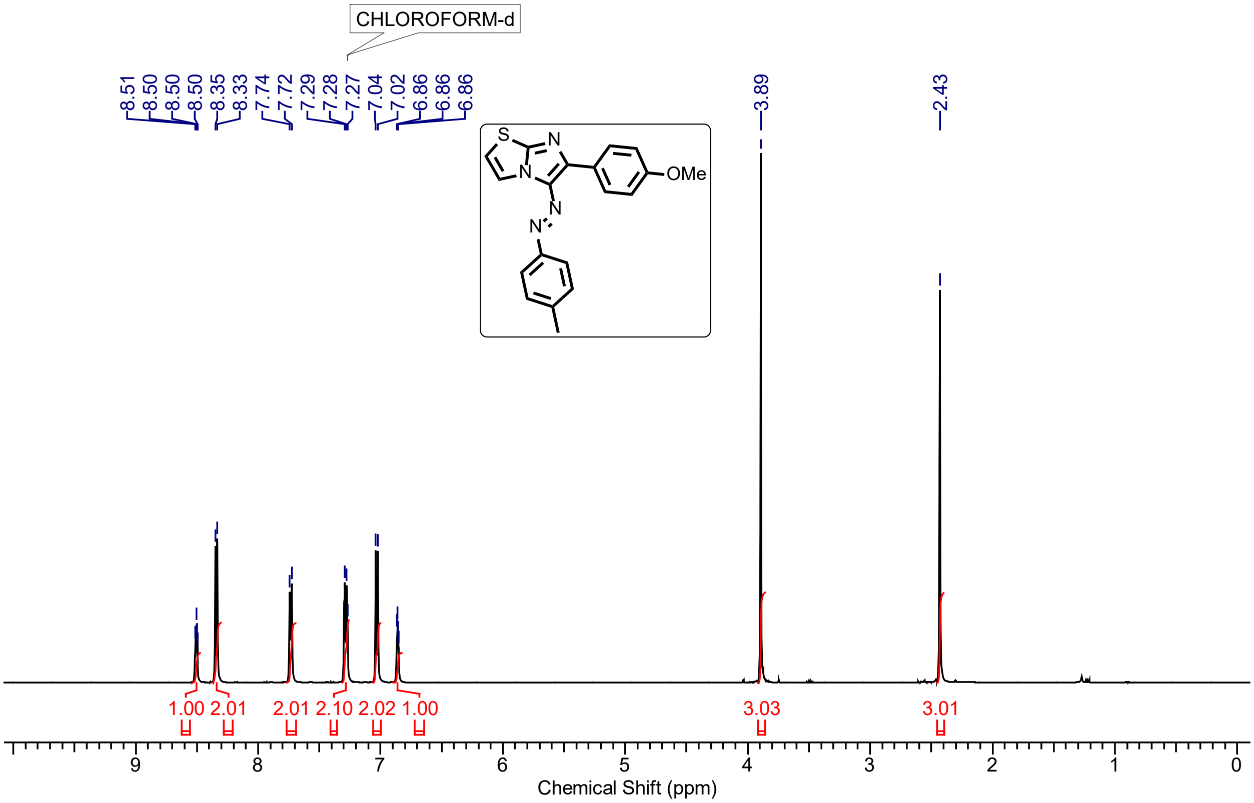

Supplementary Figure 28: ^{13}C-NMR spectra of (E)-6-(4-Methoxyphenyl)-5-(p-tolyldiazenyl)imidazo[2,1-b]thiazole

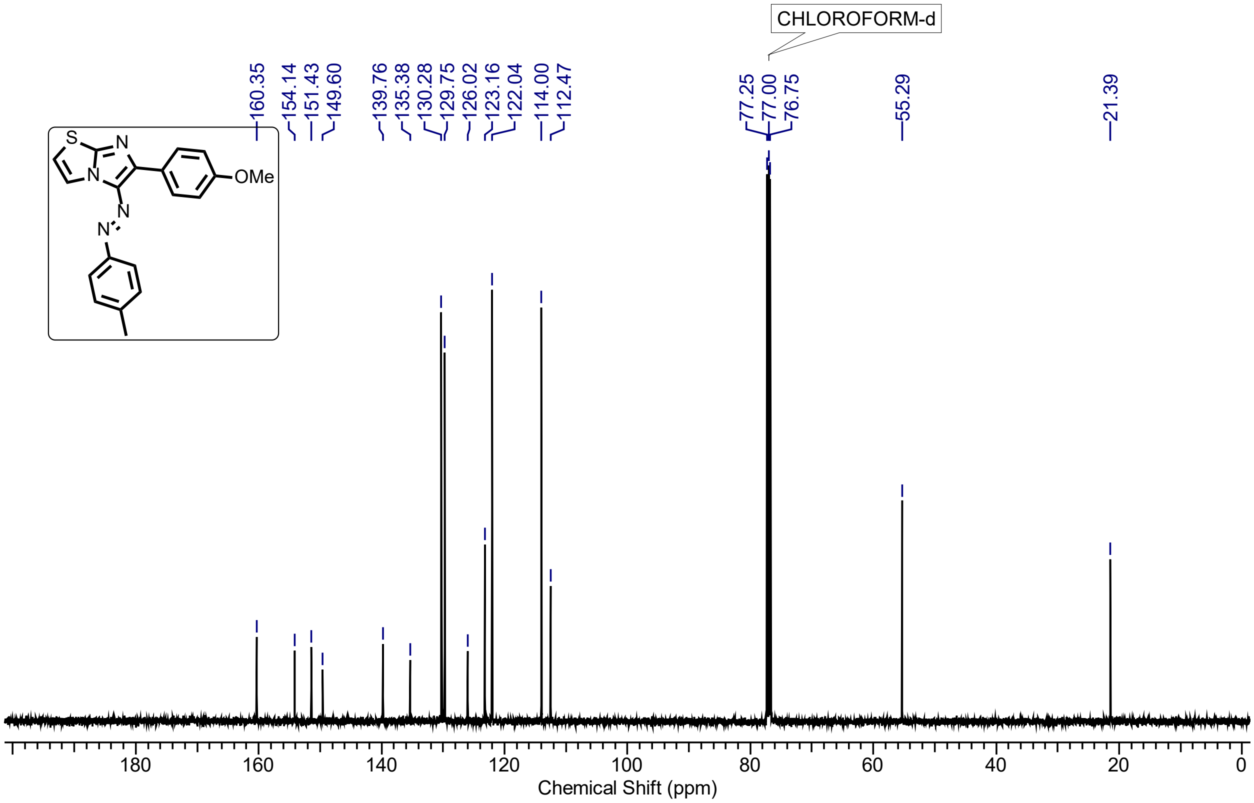

Supplementary Figure 29: ^{1}H-NMR spectra of (E)-1-(Phenyldiazenyl)naphthalen-2-ol

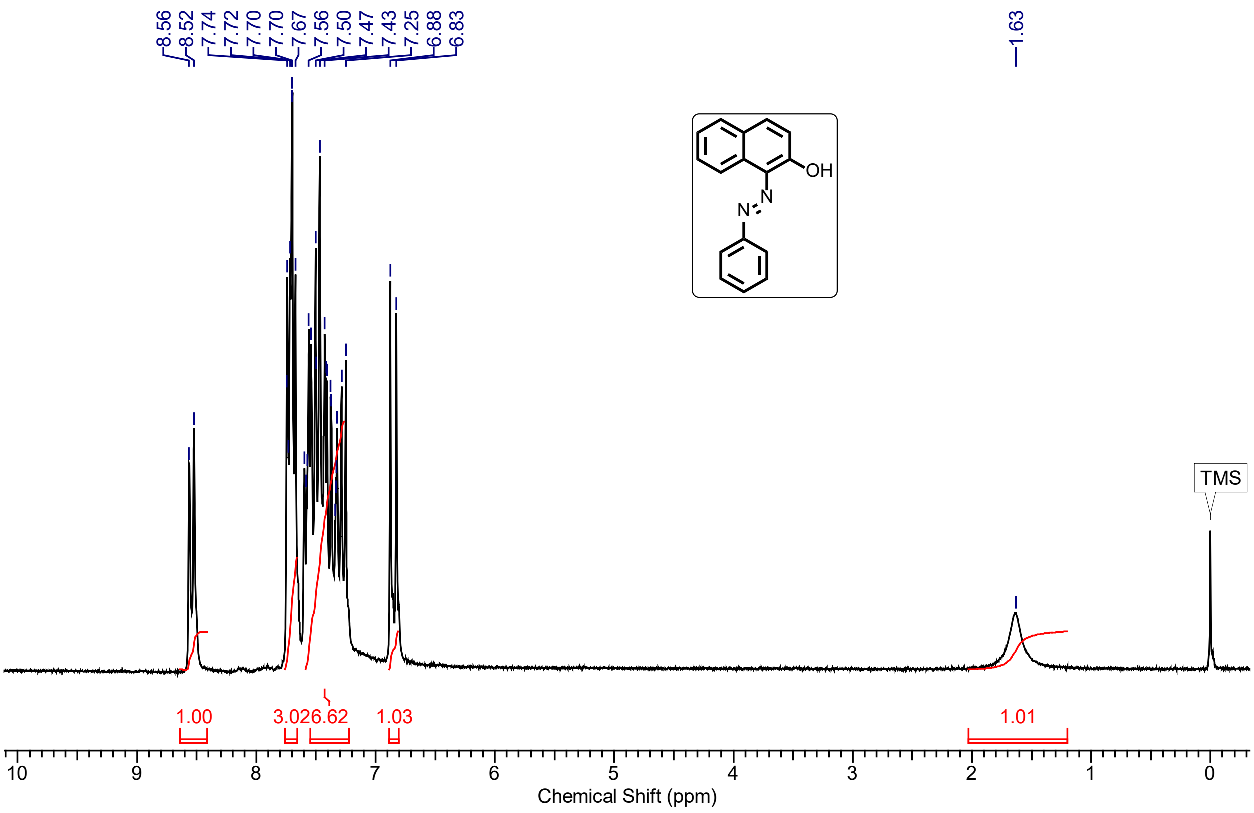

Supplementary Figure 30: ^{13}C-NMR spectra of (E)-1-(Phenyldiazenyl)naphthalen-2-ol

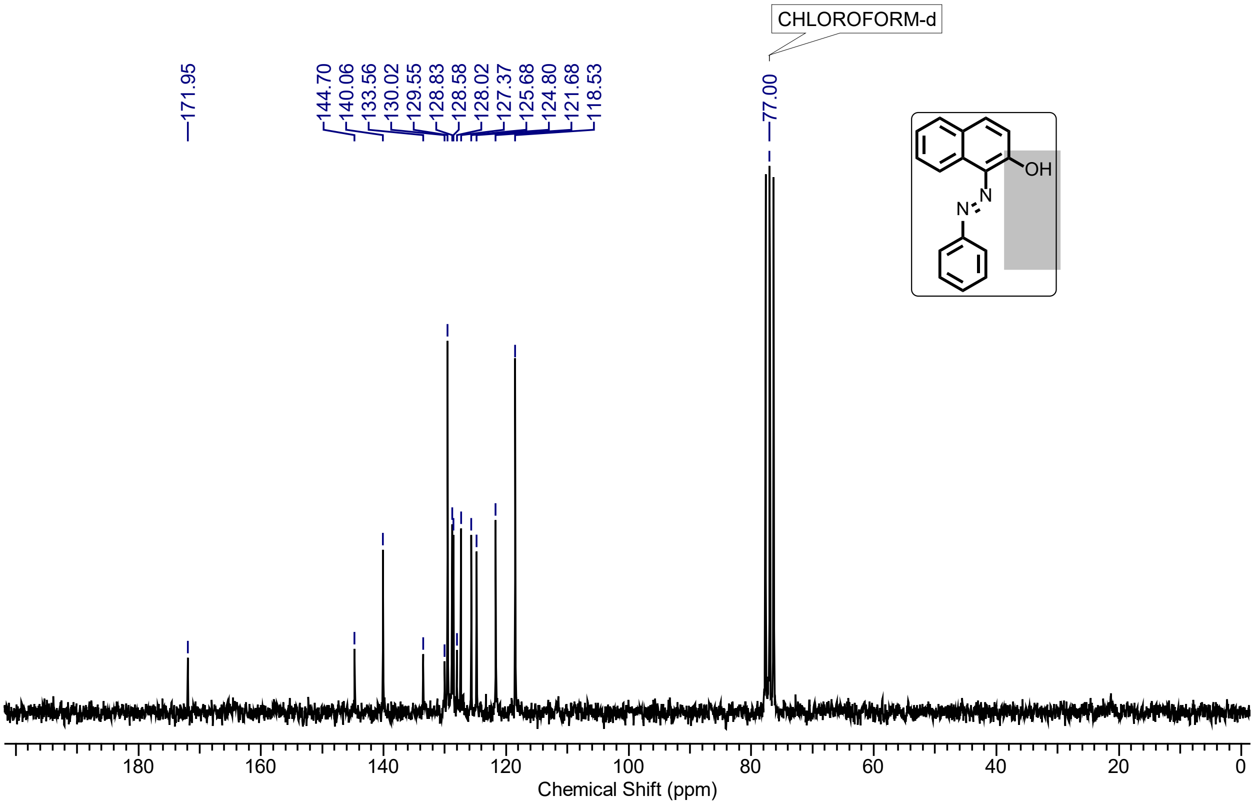

Supplementary Figure 31: ^{1}H-NMR spectra of (E)-1-(p-Tolyldiazenyl)naphthalen-2-ol

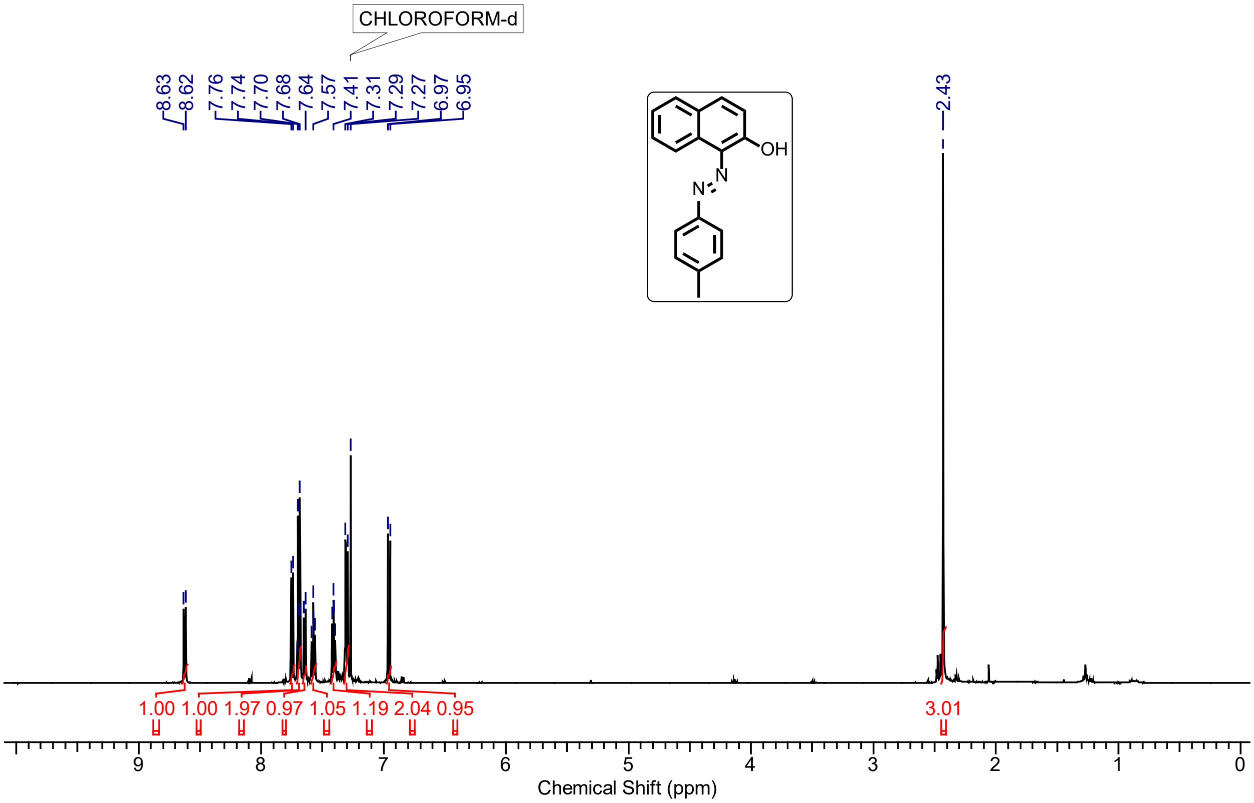

Supplementary Figure 32: ^{13}C-NMR spectra of (E)-1-(p-Tolyldiazenyl)naphthalen-2-ol

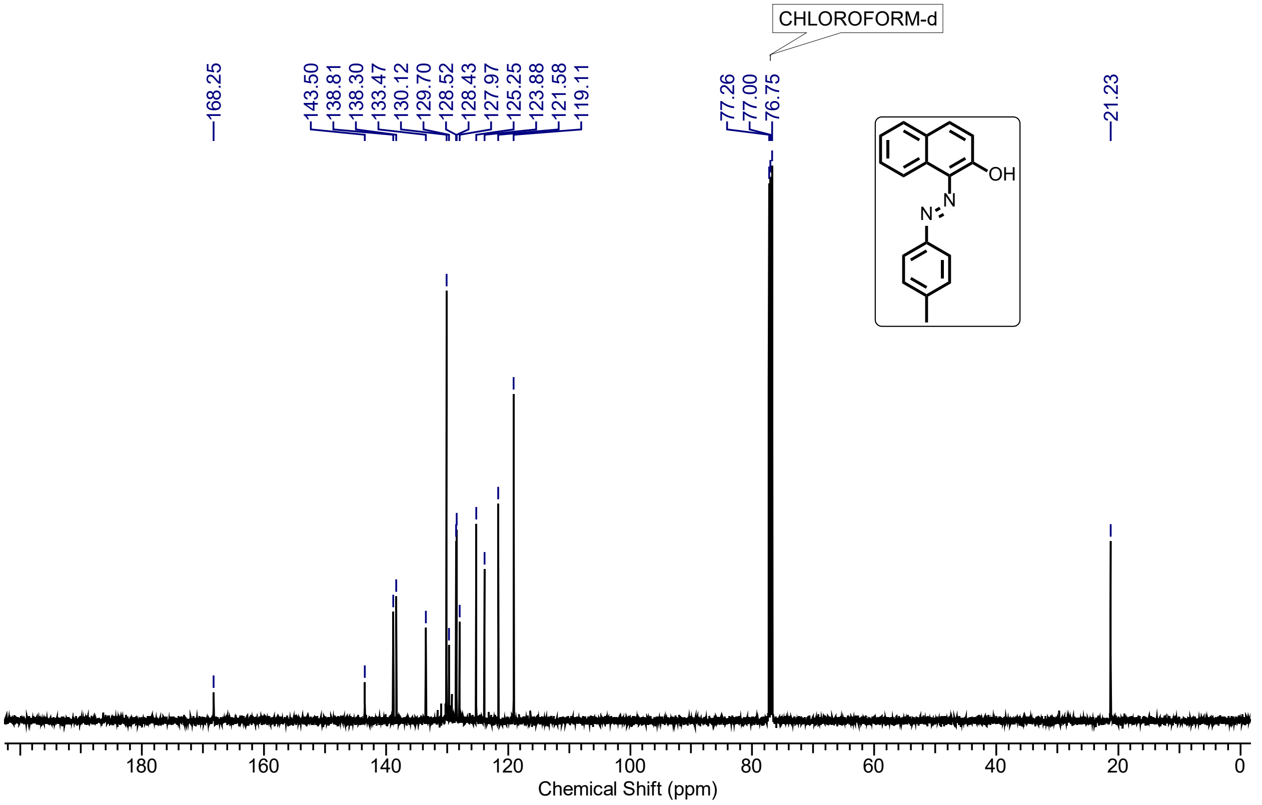

Supplementary Figure 33: ^{1}H-NMR spectra of Iodobenzene

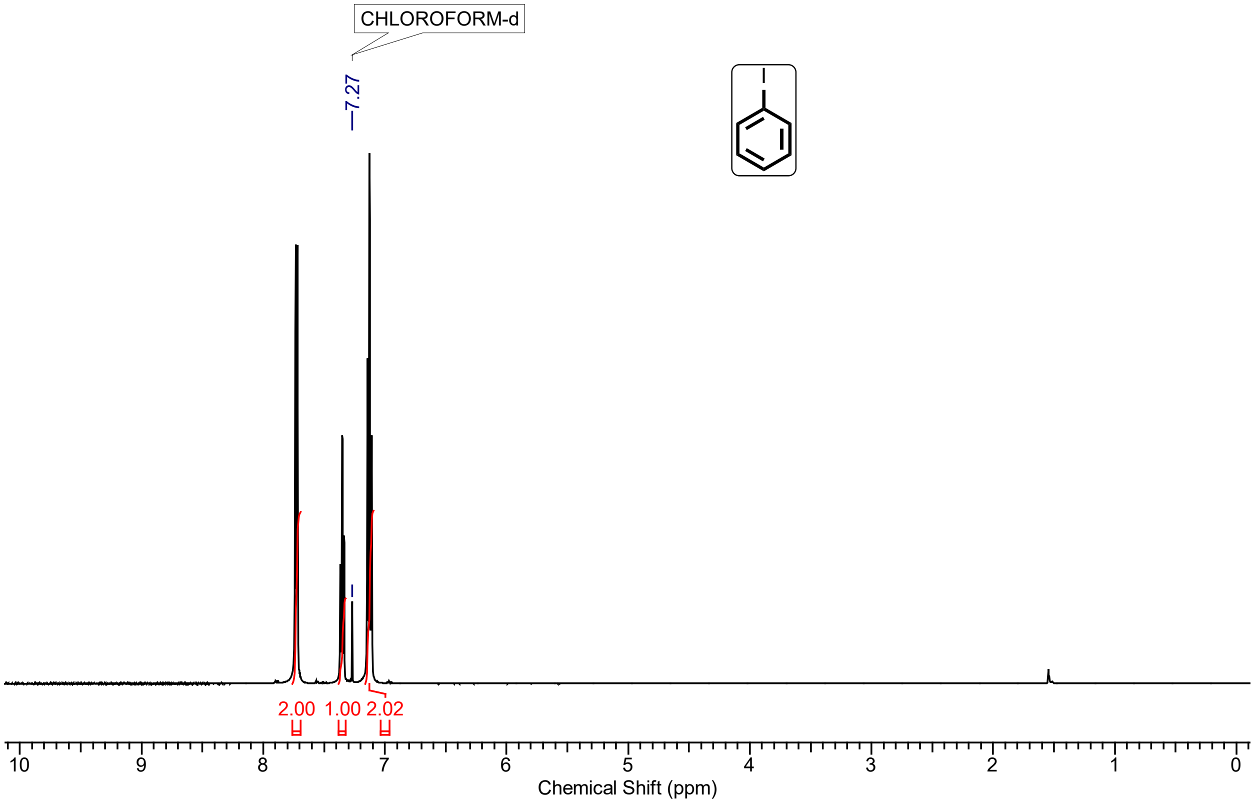

Supplementary Figure 34: ^{13}C-NMR spectra of Iodobenzene

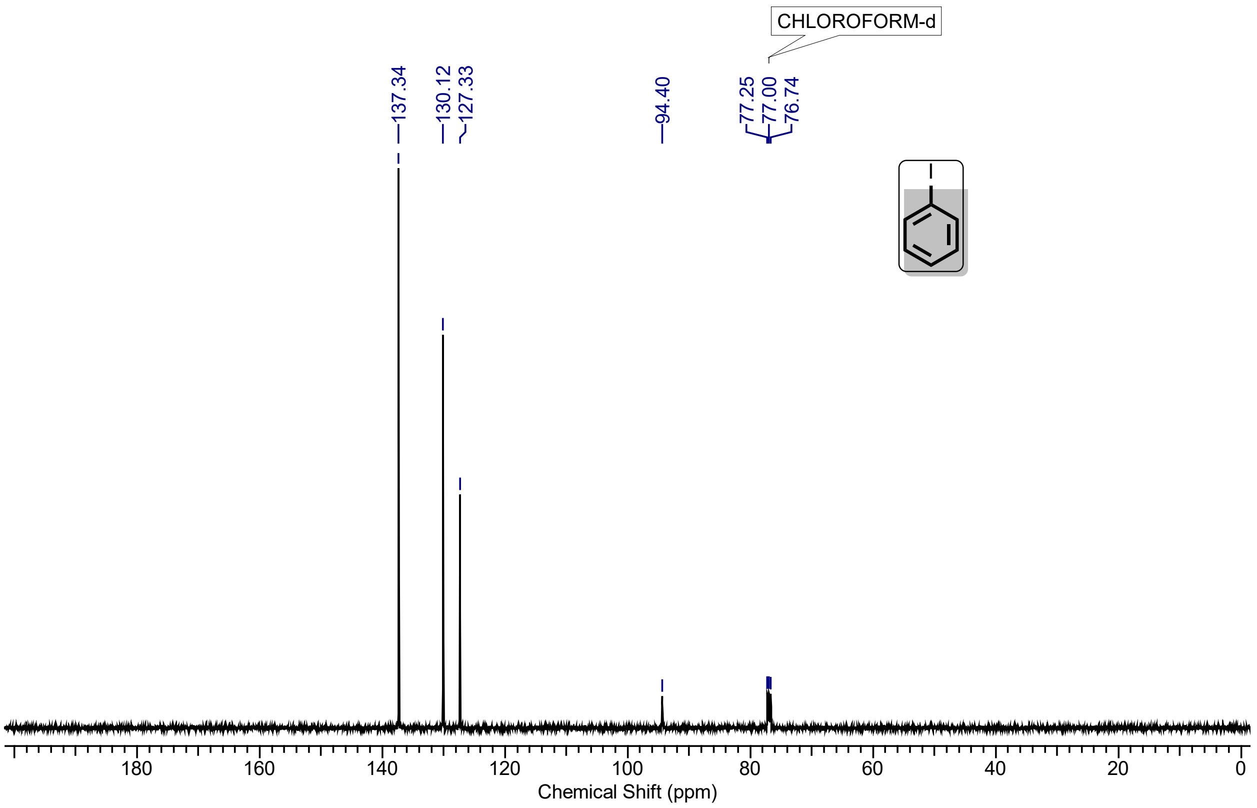

Supplementary Figure 35: ^{1}H-NMR spectra of 1-Iodo-4-methylbenzene

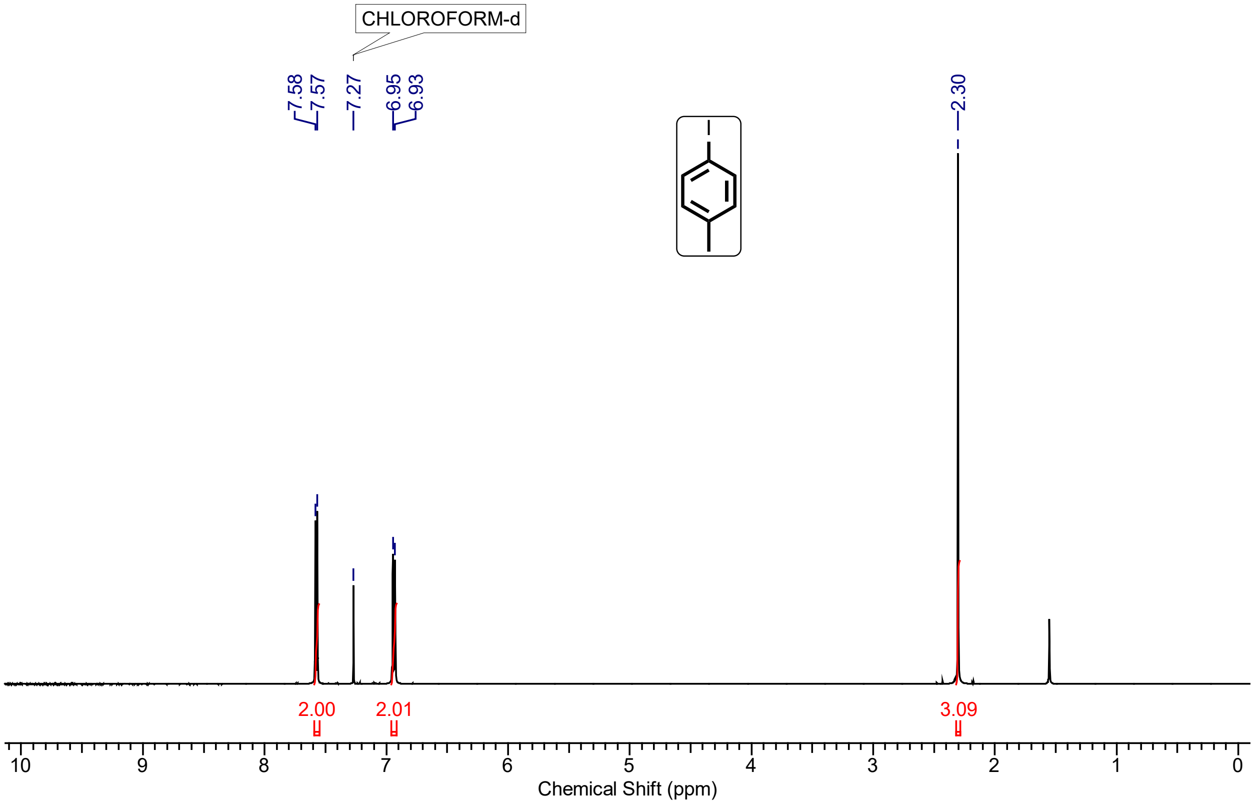

Supplementary Figure 36: ^{13}C-NMR spectra of 1-Iodo-4-methylbenzene

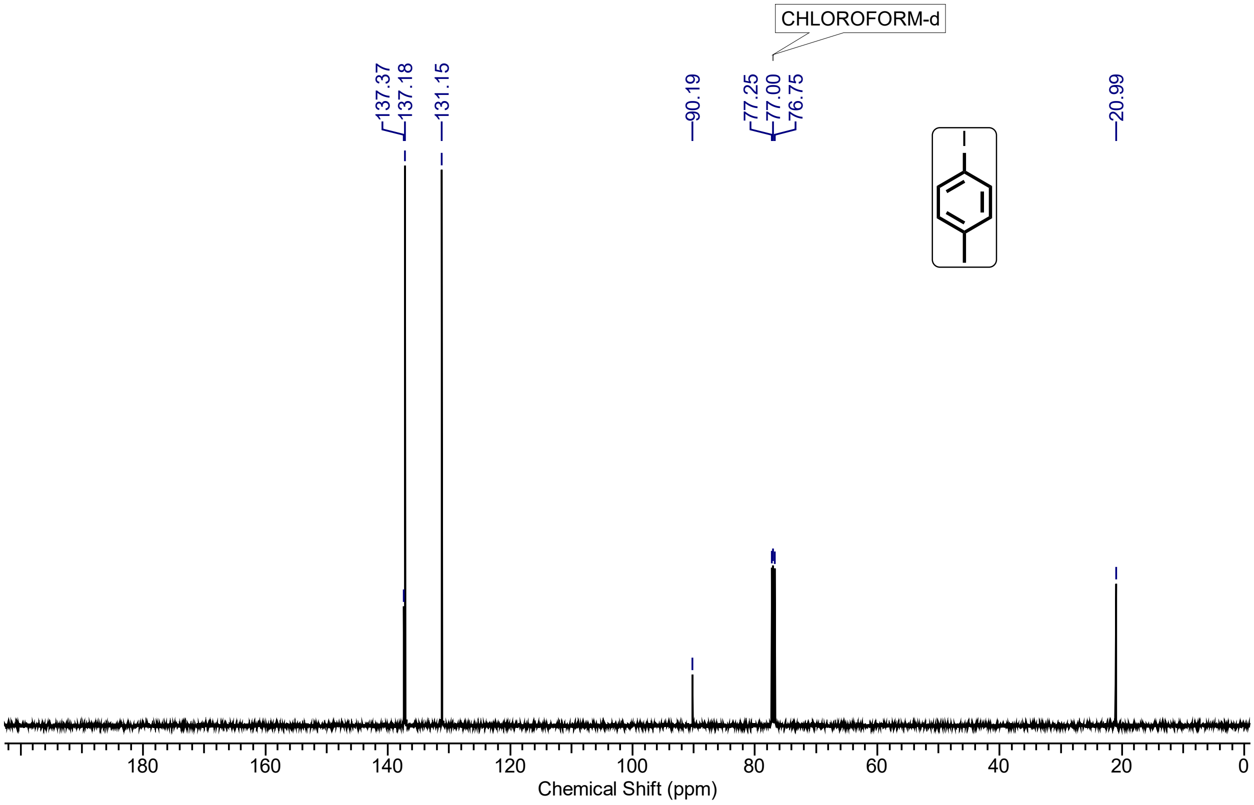

Supplementary Figure 37: ^{1}H-NMR spectra of Chlorobenzene

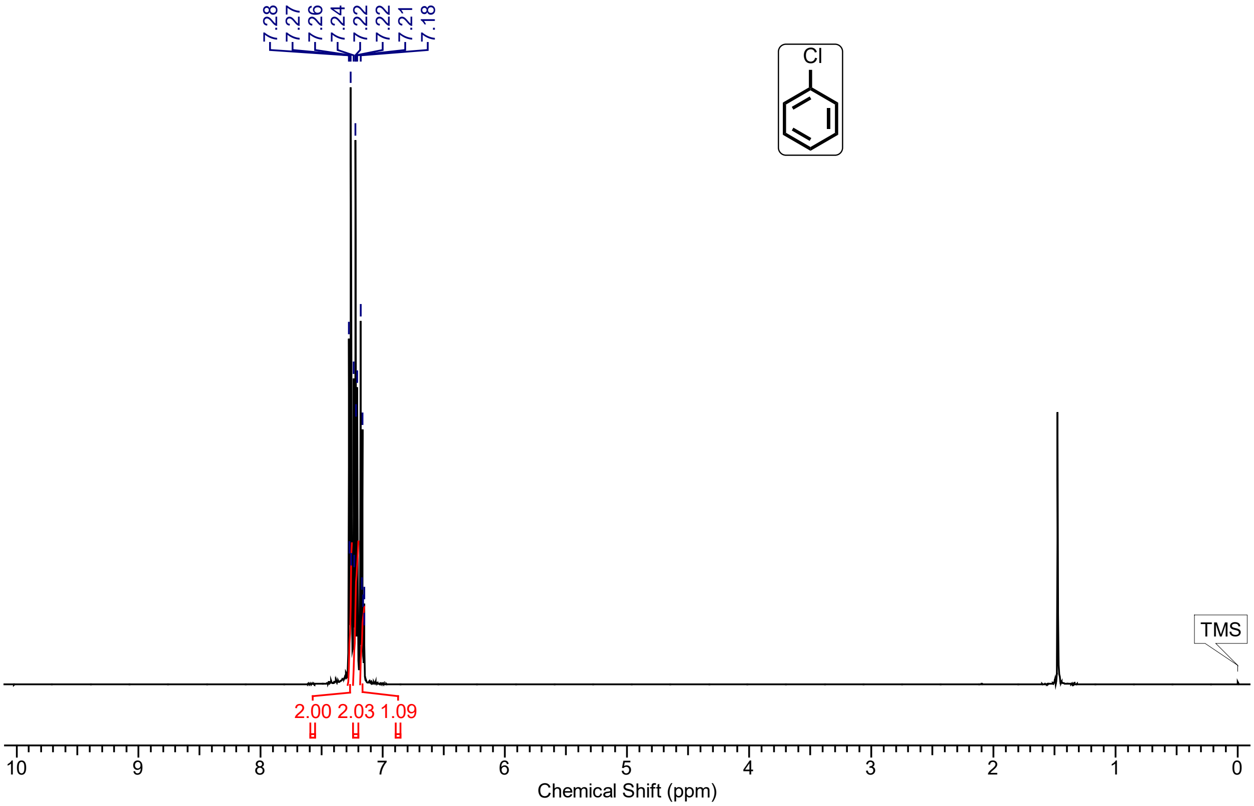

Supplementary Figure 38: ^{13}C-NMR spectra of Chlorobenzene

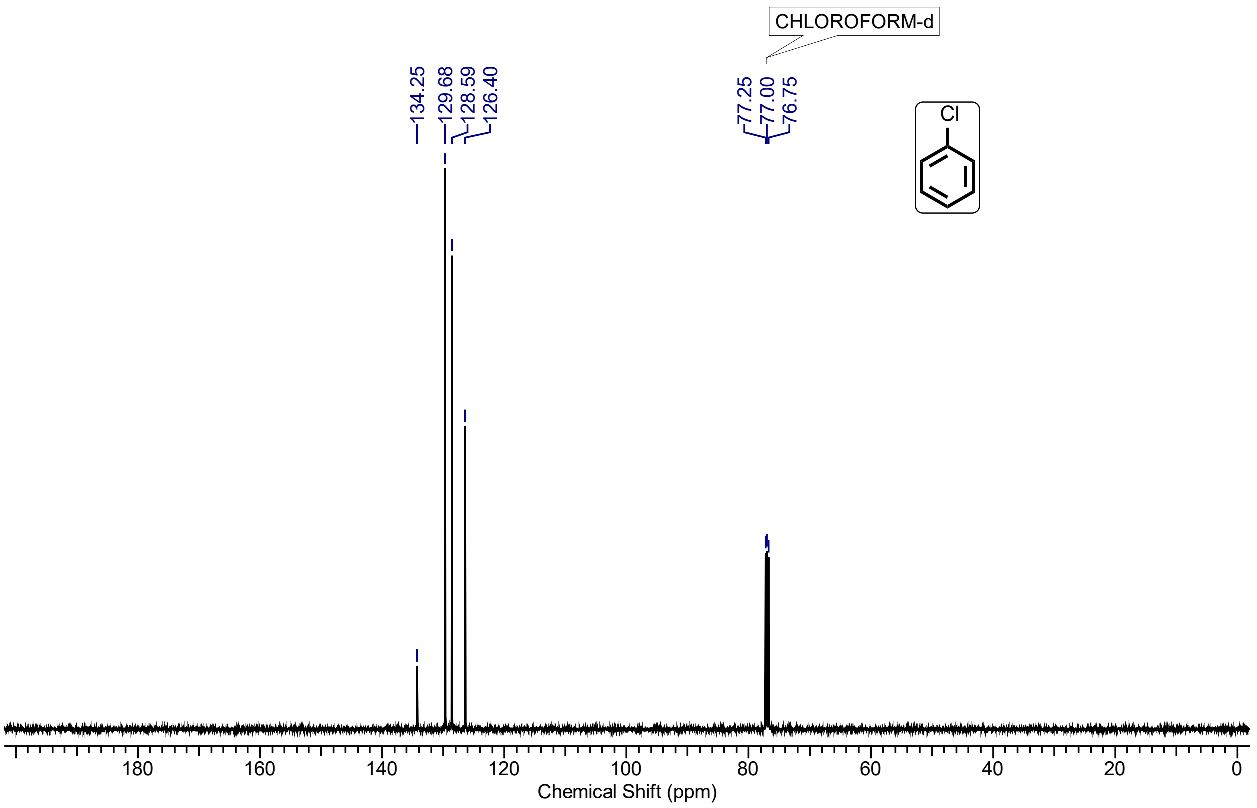

Supplementary Figure 39: ^{1}H-NMR spectra of 1-Chloro-4-methylbenzene

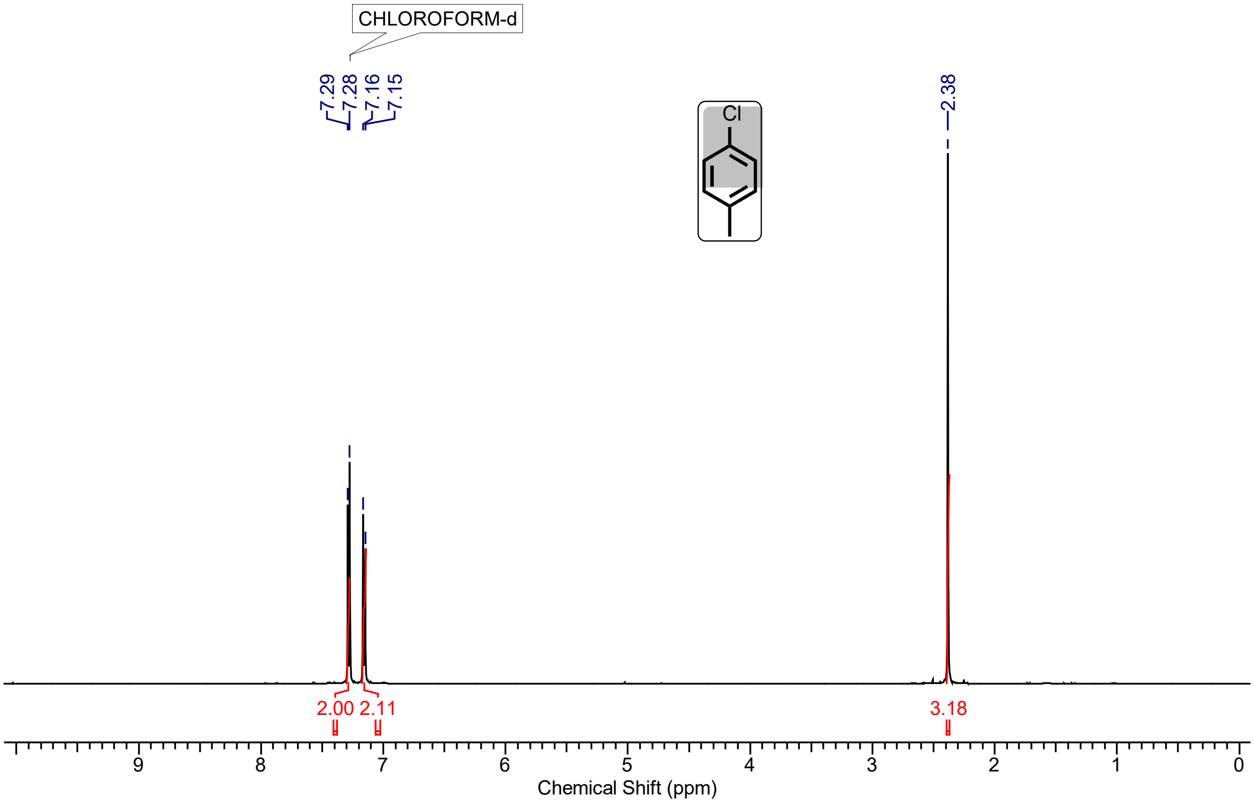

Supplementary Figure 40: ^{13}C-NMR spectra of 1-Chloro-4-methylbenzene

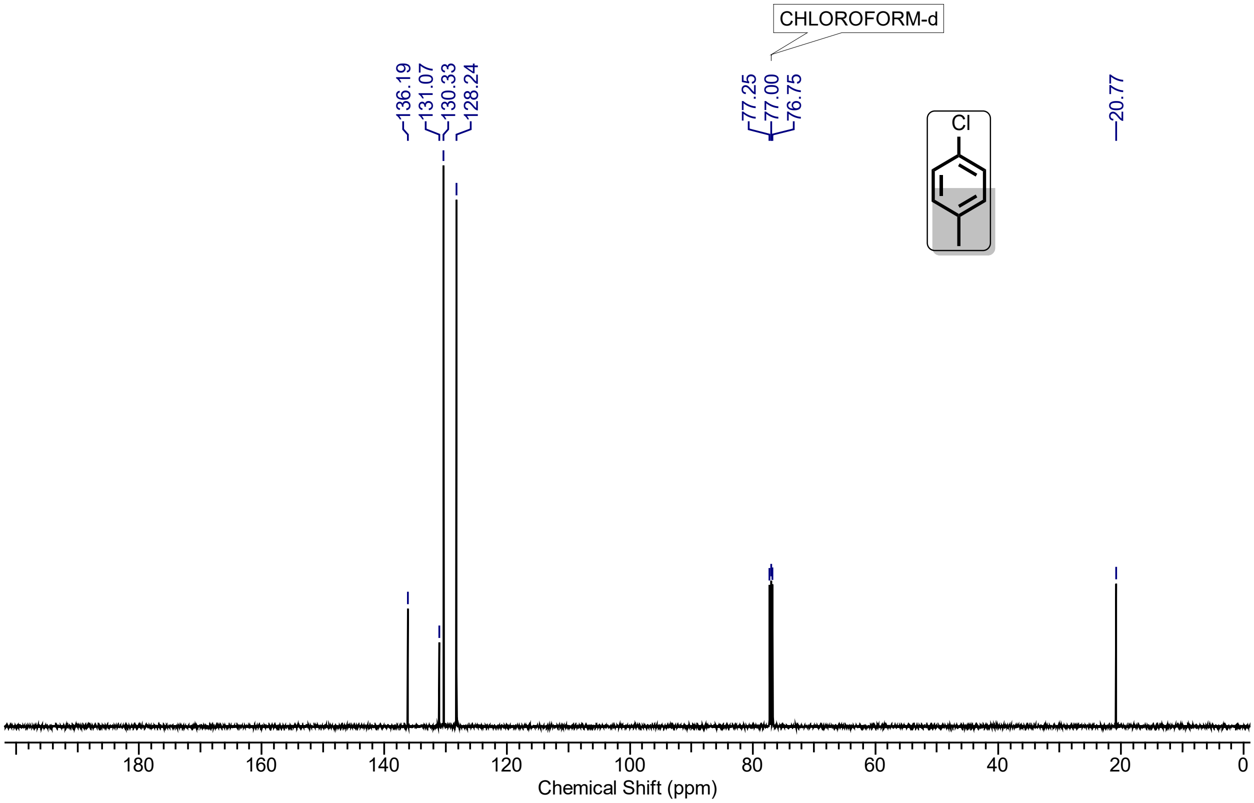

Supplementary Figure 41: ^{1}H-NMR spectra of 2-Phenylfuran

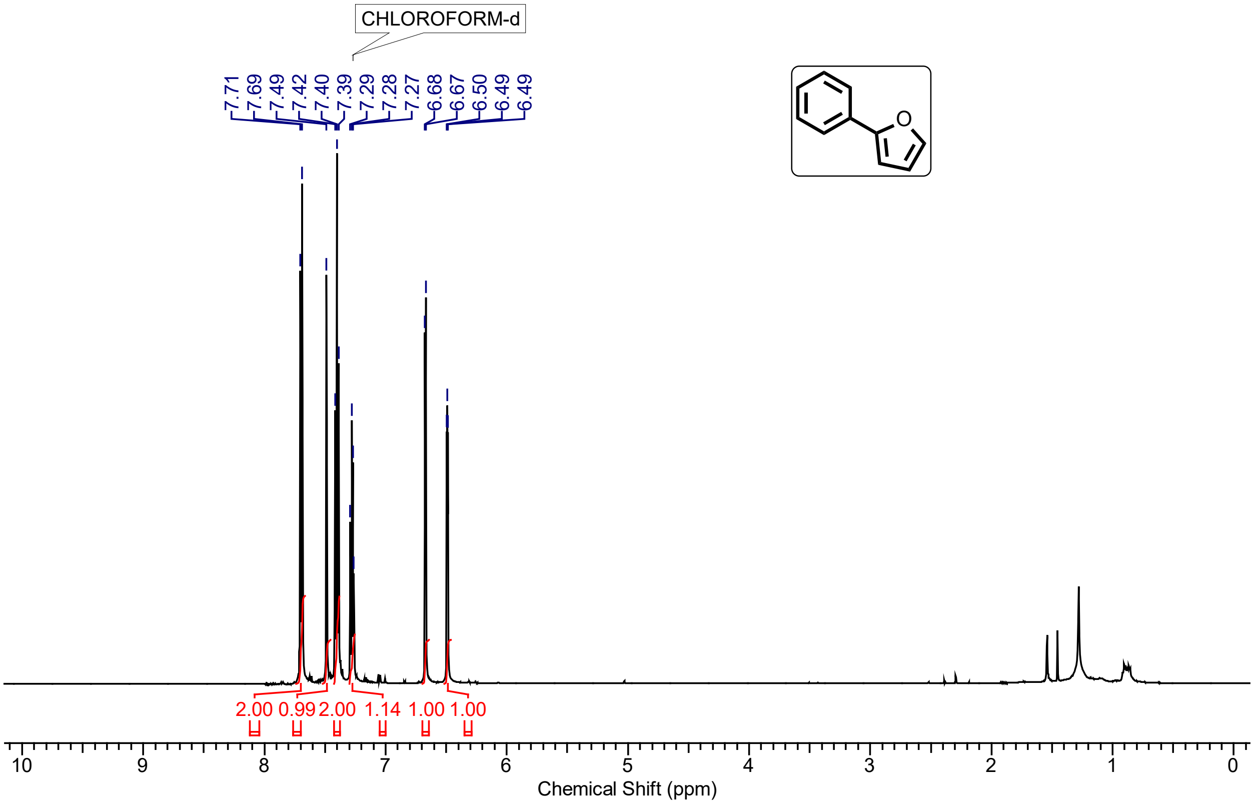

Supplementary Figure 42: ^{13}C-NMR spectra of 2-Phenylfuran

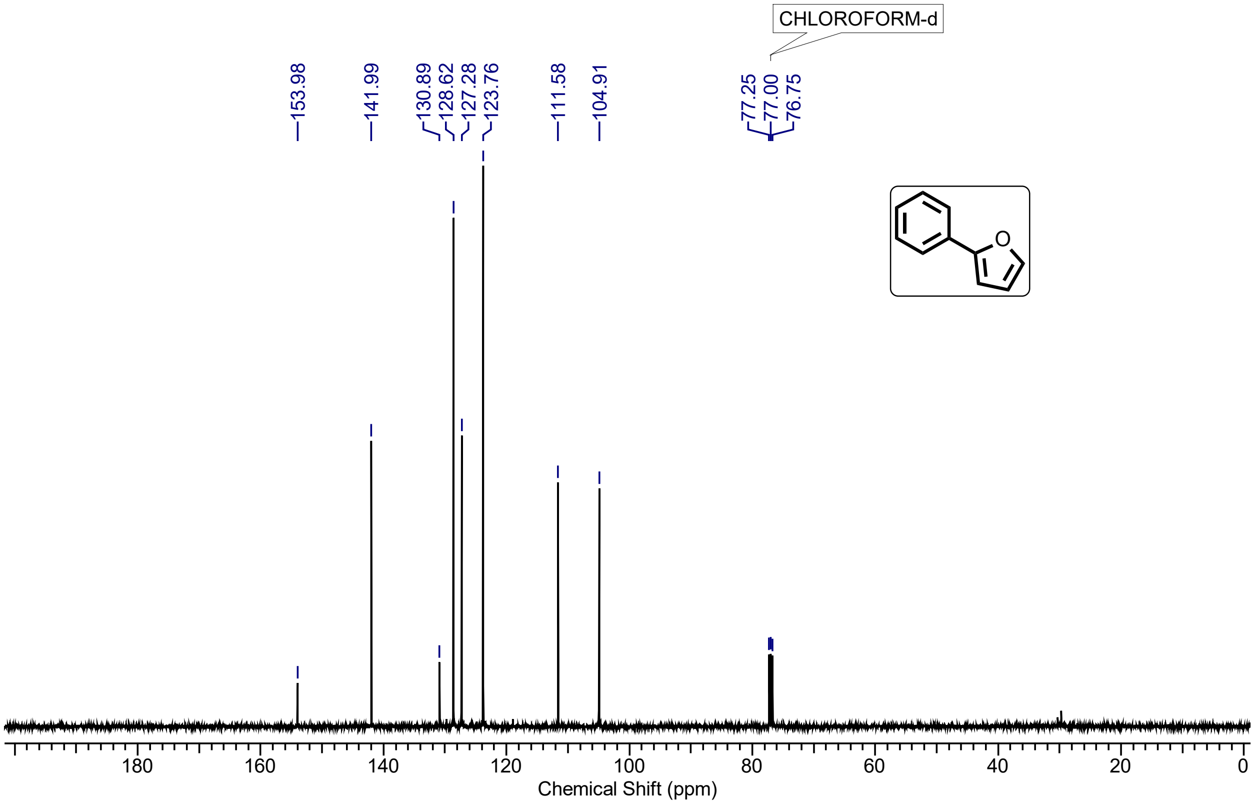

Supplementary Figure 43: ¹H-NMR spectra of 2-(p-Tolyl)furan

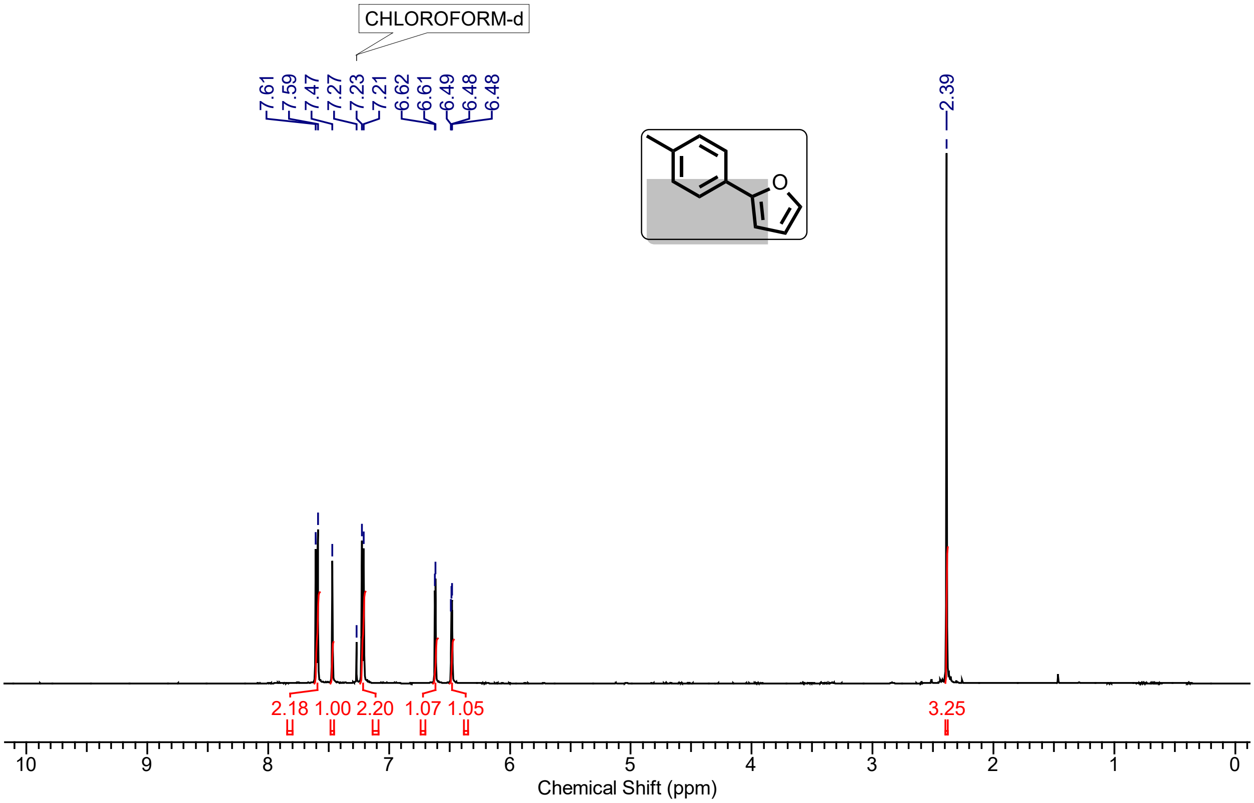

Supplementary Figure 44: ^{13}C-NMR spectra of 2-(p-Tolyl)furan

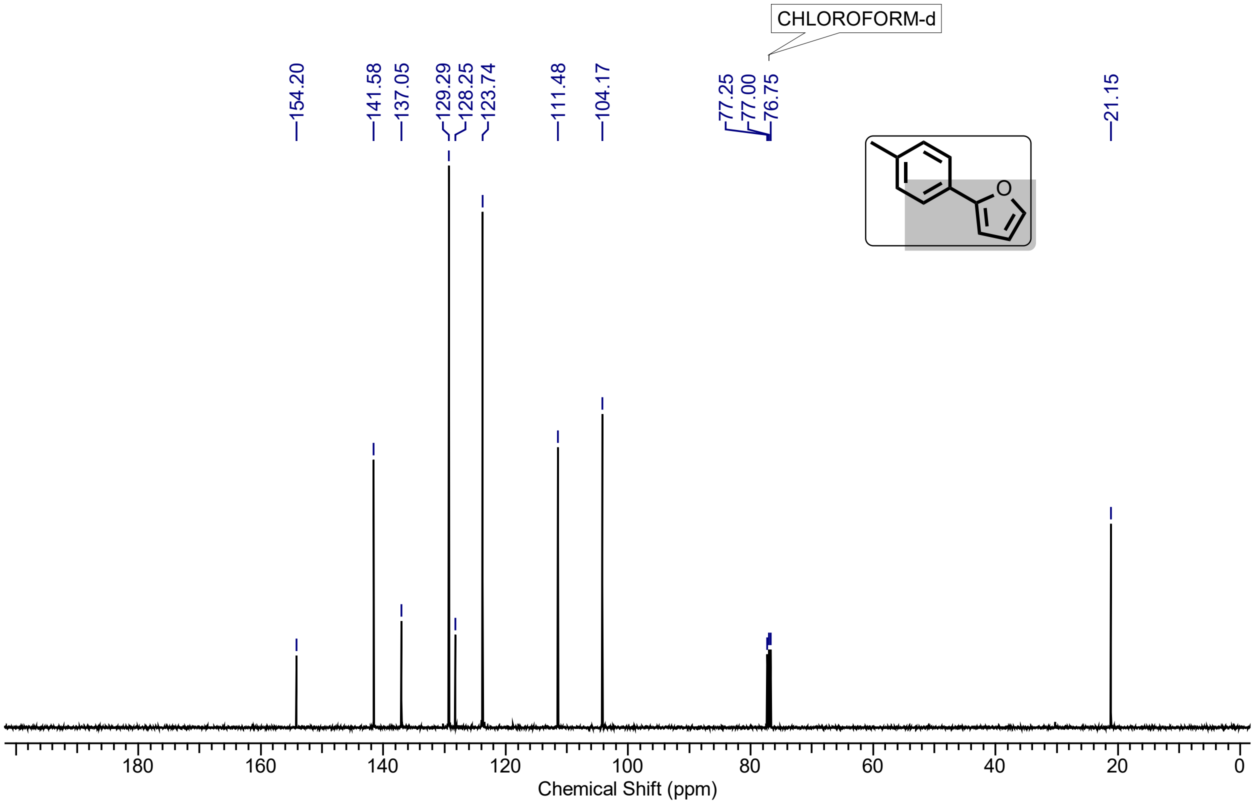

Supplementary Figure 45: ^{1}H-NMR spectra of Phenyl(p-tolyl)sulfane

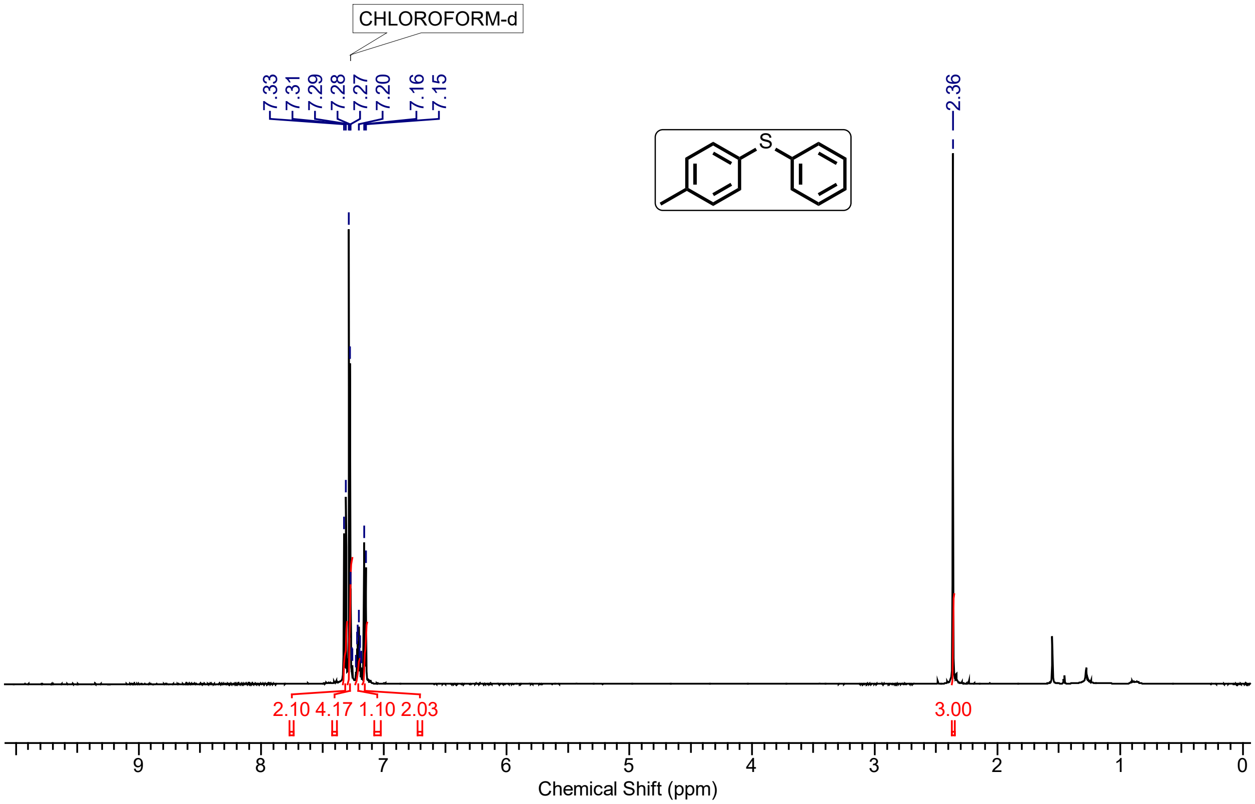

Supplementary Figure 46: ^{13}C-NMR spectra of Phenyl(p-tolyl)sulfane

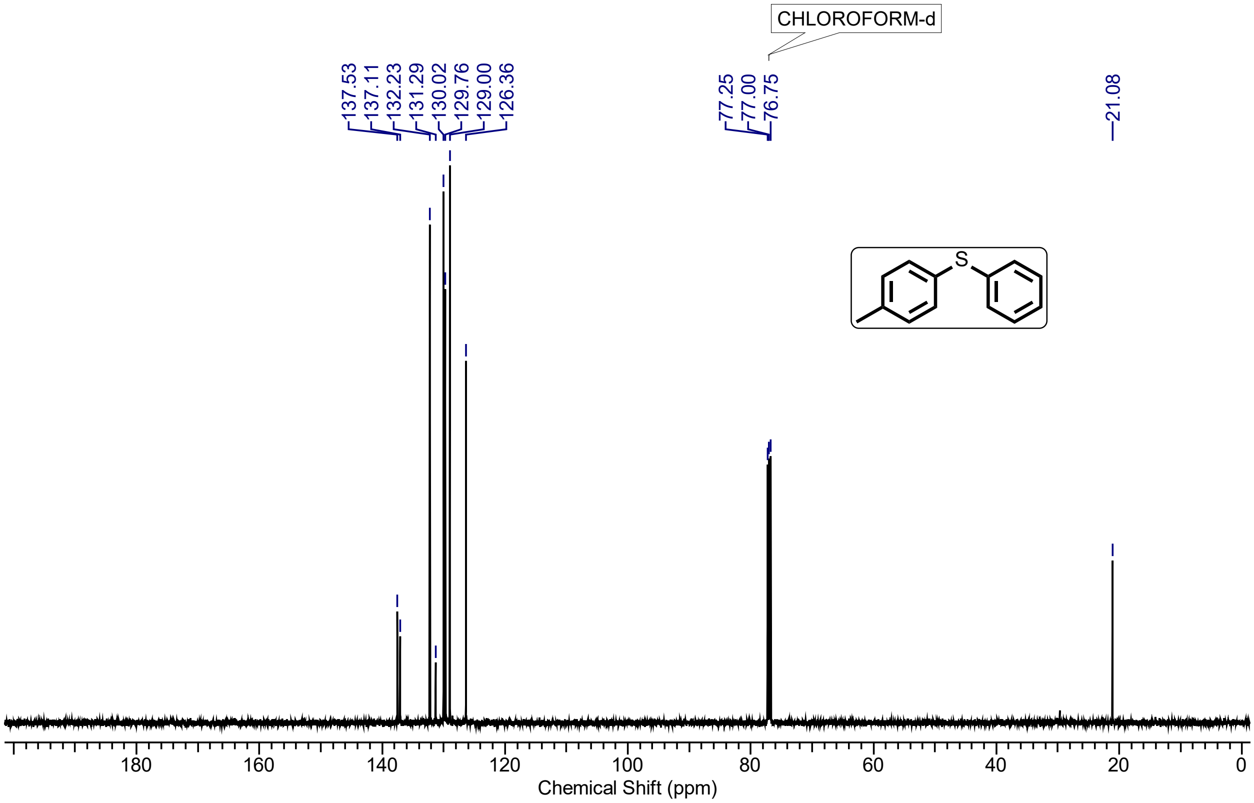

Supplementary Figure 47: ^{1}H-NMR spectra of Di-p-tolylsulfane

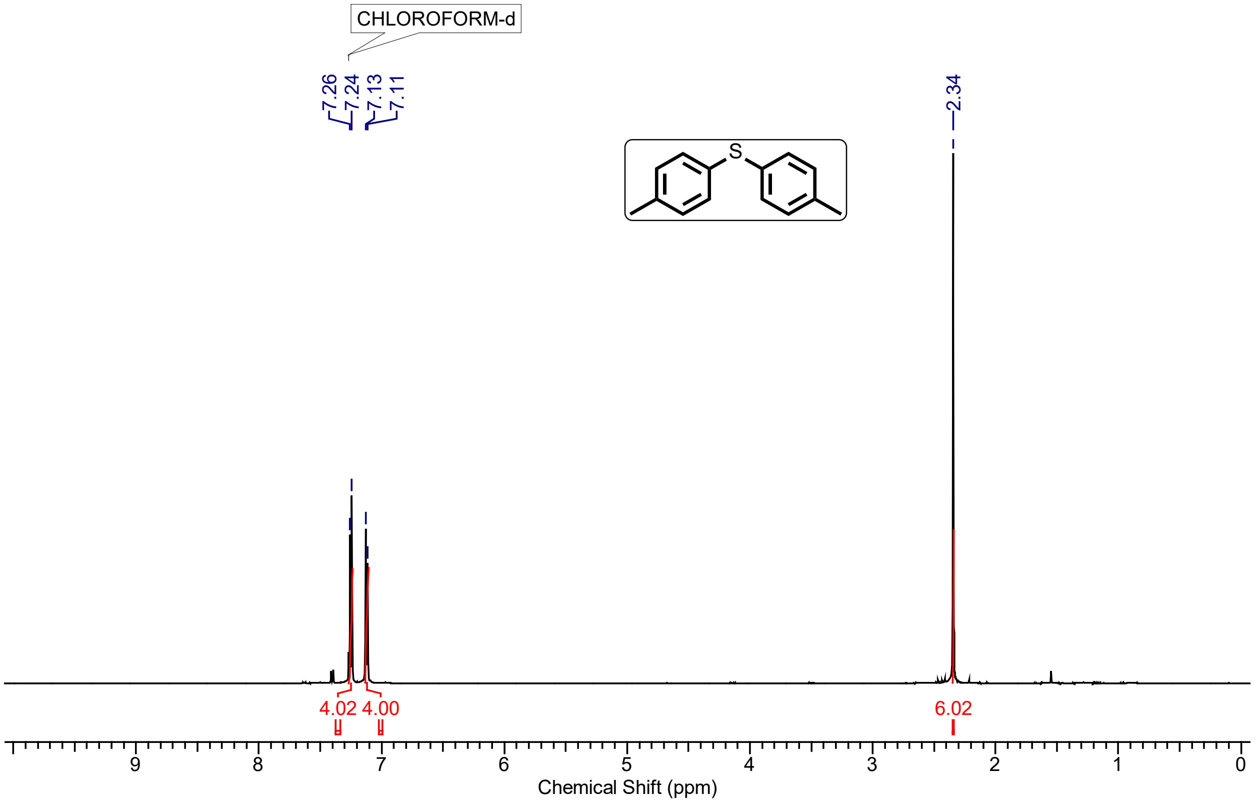

Supplementary Figure 48: ^{13}C-NMR spectra of Di-p-tolylsulfane

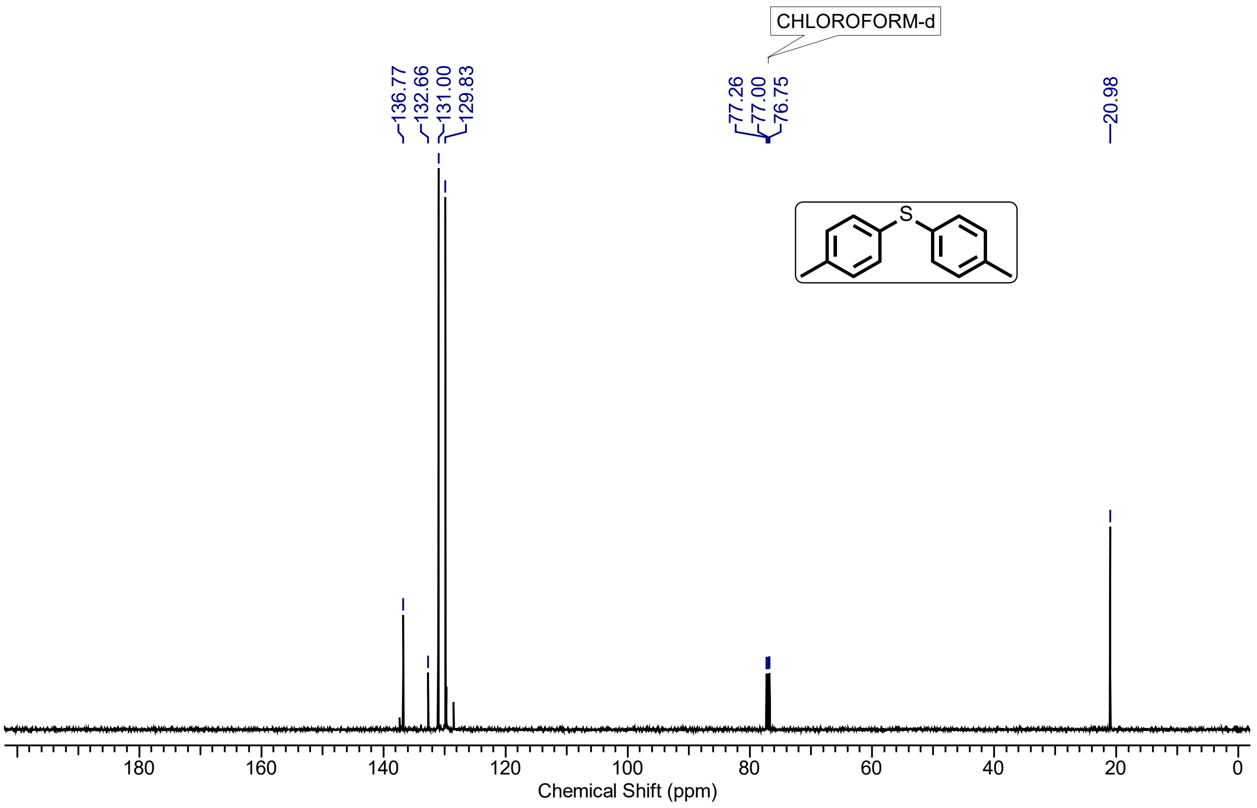

Supplementary Figure 49: ^{1}H-NMR spectra of Azidobenzene

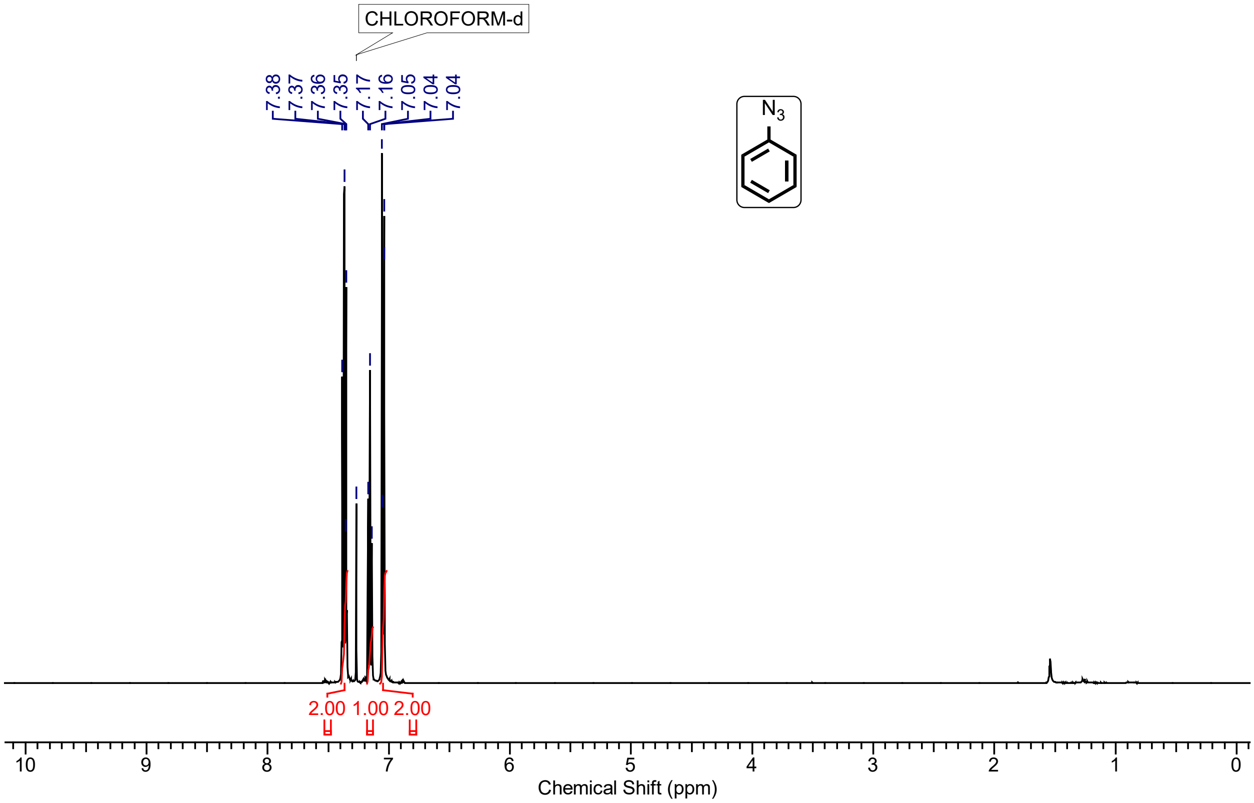

Supplementary Figure 50: ^{13}C-NMR spectra of Azidobenzene

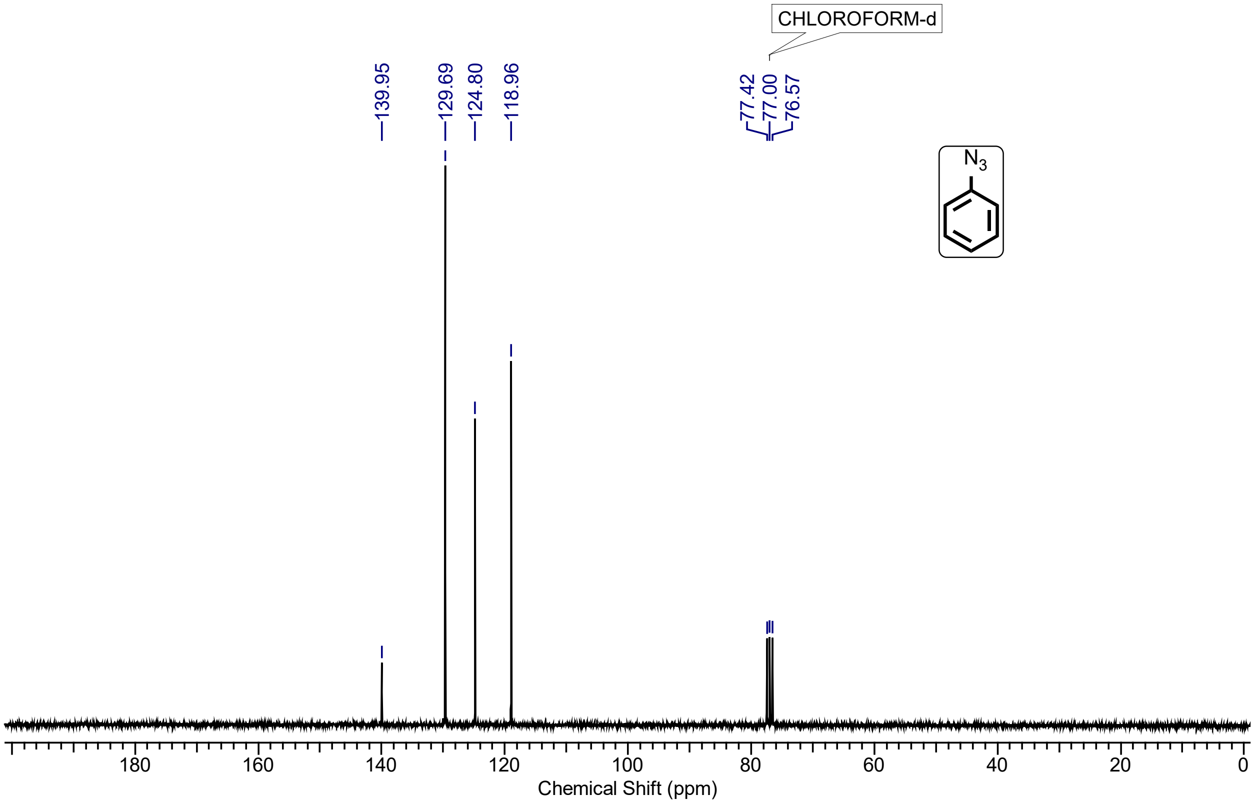

Supplementary Figure 51: ^{1}H-NMR spectra of 1-Azido-4-methylbenzene

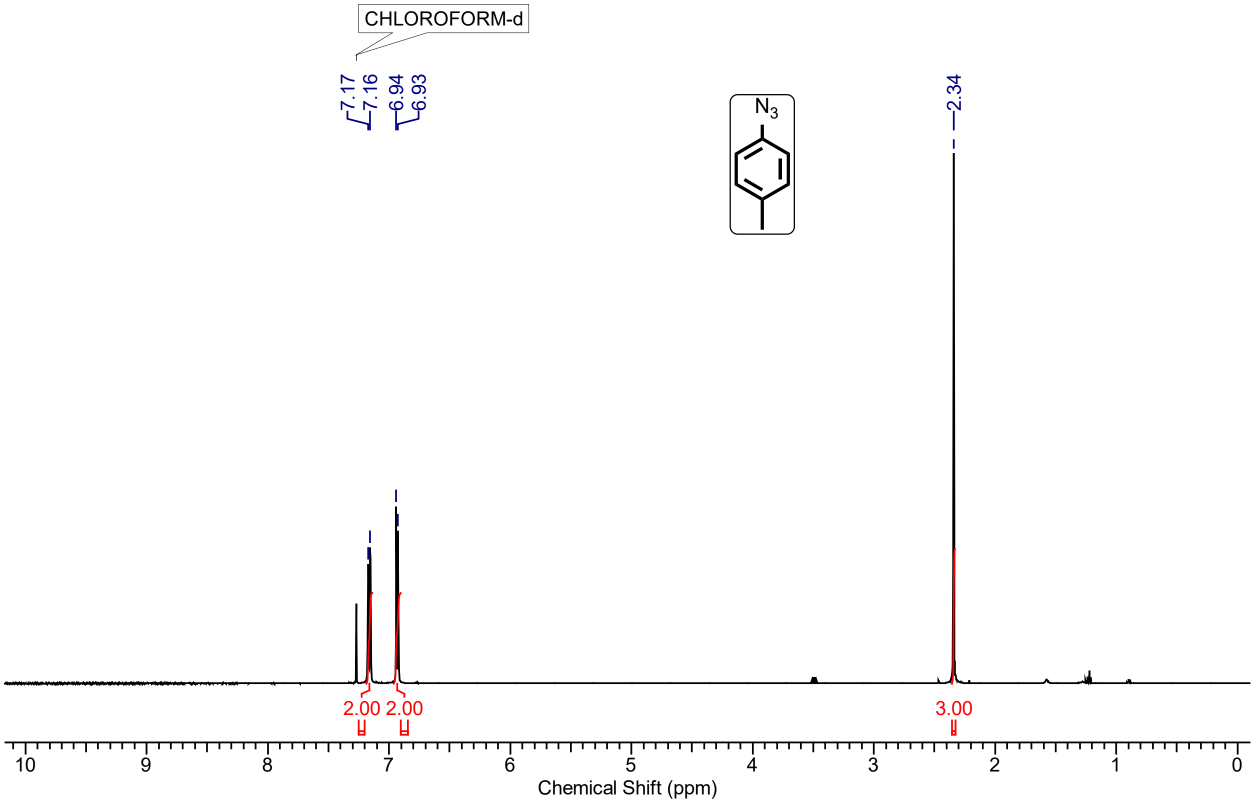

Supplementary Figure 52: ^{13}C-NMR spectra of 1-Azido-4-methylbenzene

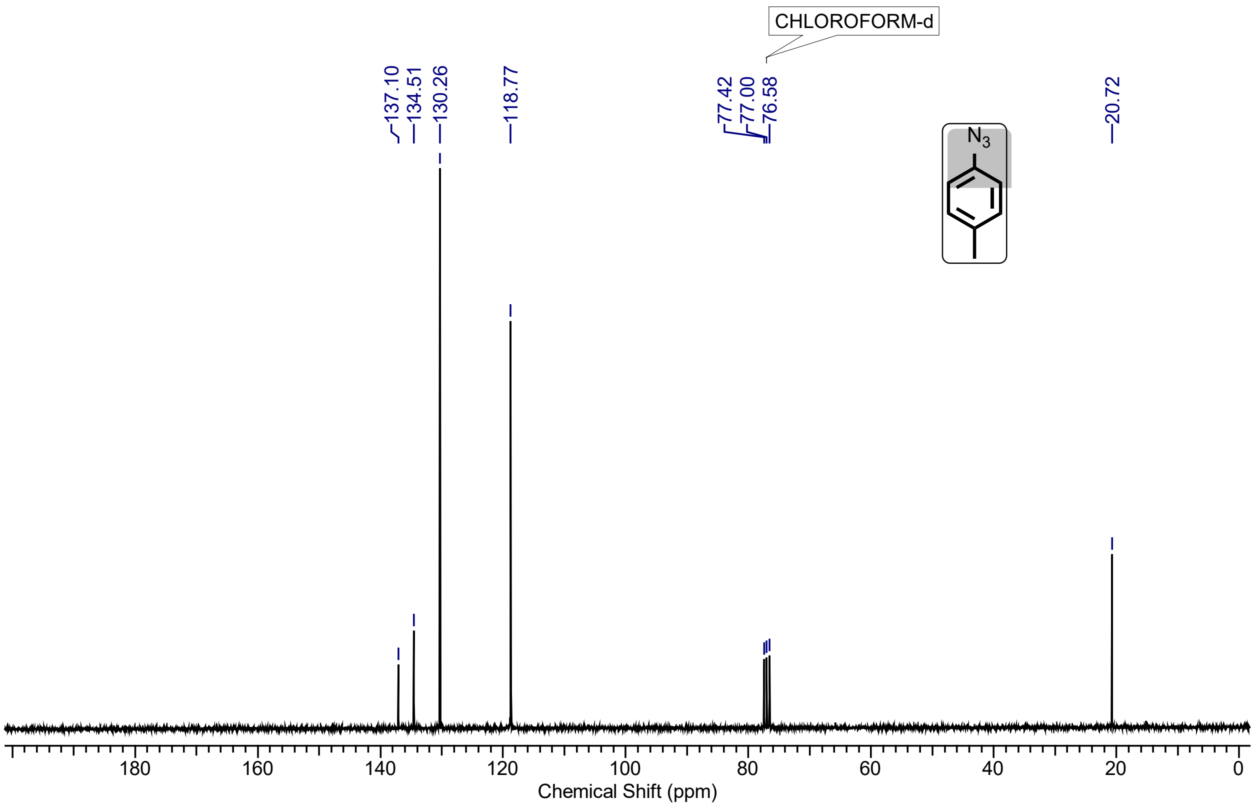

Supplement: Supplementary file 1 — Supplementary Information [file 42004_2021_490_MOESM1_ESM.pdf]
