# Supplementary material for: Flow parallel synthesizer for multiplex synthesis of aryl diazonium libraries via efficient parameter screening
Source: Commun Chem. 2021 Apr 15;4:53. doi: 10.1038/s42004-021-00490-6 (PMC9814388; doi:10.1038/s42004-021-00490-6)
Supplement: Supplementary file 2 — Description of Additional Supplementary Files [file 42004_2021_490_MOESM2_ESM.pdf]

Description of Additional Supplementary Files

File Name: Supplementary Video 1
Description: Video showing the experimental process to check the effect of the presence or absence of diazonium salt on the distribution performance. The distribution performance was compared when only DMSO was flowed with a total flow of 10.56 ml min-1 and DMSO with diazonium salt was flowed with a total flow of 10.56 ml min-1.

File Name: Supplementary Video 2
Description: Video showing concentration screening process of Azo-dye. Azo-dye causes clogging in high concentration or low flow conditions after system stabilization. Even if single or several capillaries are clogged, the remaining capillaries have uniform flow behavior.

File Name: Supplementary Video 3
Description: Video showing the process of the multiplex synthesis of 24 compound libraries.
